# Evaluating UAV-based phenotyping strategies for *Megathyrsus maximus*

**DOI:** 10.3389/fpls.2026.1798414

**Published:** 2026-04-22

**Authors:** Guilherme Francio Niederauer, Alexandre Hild Aono, Mateus Figueiredo Santos, Celina de Medeiros Ragalzi, Liana Jank, Anete Pereira de Souza

**Affiliations:** 1 Center for Plant Molecular Breeding (CeM²P), University of Campinas (UNICAMP), Campinas, SP, Brazil; 2 Center for Molecular Biology and Genetic Engineering (CBMEG), University of Campinas (UNICAMP), Campinas, SP, Brazil; 3 Department of Plant Breeding, Swedish University of Agricultural Sciences (SLU), Alnarp, Sweden; 4 Embrapa Beef Cattle, Empresa Brasileira de Pesquisa Agropecuária (EMBRAPA), Campo Grande, MS, Brazil; 5 Department of Plant Biology, Biology Institute (IB), University of Campinas (UNICAMP), Campinas, SP, Brazil

**Keywords:** digital traits, forage breeding, high-throughput phenotyping, machine learning, remote sensing, trait prediction

## Abstract

Effective high-throughput phenotyping is crucial for modern plant breeding, yet the optimal image acquisition parameters for UAV-based systems in forage crops remain poorly defined. We optimized UAV-based phenotyping methods for a *Megathyrsus maximus* biparental population, examining how ground sampling distance (GSD), environment, and harvest date affect the accuracy of RGB-derived digital traits in predicting yield and canopy height. Machine learning algorithms and mixed model analyses were applied to evaluate predictive power and heritability. Pixel count and Haralick's entropy showed strong correlations with conventional yield measurements, particularly in Environment 2, while most vegetative indices were poor predictors. Integrating machine learning substantially enhanced predictive power for green and dry matter yield (r > 0.80). For canopy height, machine learning models achieved correlations of 0.71 with ground truth measurements despite weak pairwise correlations. Mixed model analysis revealed high broad-sense heritability (0.7 < *H*^2^ < 0.87) for yield traits, pixel count, and entropy, while vegetative indices and canopy height showed greater environmental susceptibility. Moderate GSD resolutions (0.5–1.0 cm) consistently outperformed both very high (0.27 cm) and very low (1.5 cm) resolutions. Coincidence index analysis demonstrated 80% correspondence between top genotypes ranked by pixel count and conventionally measured dry matter yield. This study provides an optimized framework for UAV-based phenotyping in *M. maximus*, demonstrating that combining advanced digital traits with machine learning accurately predicts key agronomic traits and significantly enhances genotype selection efficiency in forage breeding programs.

## 1 Introduction

Precise and efficient phenotyping is essential for advancing forage plant breeding and supporting sustainable livestock production. Although genomics and high-throughput sequencing have advanced rapidly, enabling the discovery of genetic variation underlying agronomic traits, progress in forage breeding is constrained by the lack of efficient large-scale phenotyping. This bottleneck limits genetic gain, increases costs, and hinders the effective integration of genomic information into breeding programs (Araus et al., 2018; Cheng et al., 2025). Traditional phenotyping approaches-based on manual measurements and visual assessments-are labor intensive, time-consuming, susceptible to subjective bias, and, altogether, more costly (Cheng et al., 2025). These limitations restrict the efficient evaluation of large breeding populations and slow down the selection of superior individuals, thereby reducing the overall effectiveness of forage breeding. In this context, forage breeding programs, such as those for *Megathyrsus maximus* (Jacq.) B.K. Simon & S.W.L. Jacobs, syn *Panicum maximum Jacq.* (guineagrass), a tetraploid forage species prized for its high biomass yield and nutritional quality for beef cattle, would greatly benefit from the modernization of phenotyping techniques. Although the Brazilian *M. maximus* breeding program has successfully developed several improved cultivars (e.g., ‘Tanzânia-1’, ‘Mombaça’, ‘Massai’, ‘BRS Zuri’, ‘BRS Tamani’ and ‘BRS Quênia’) (Jank et al., 2014, 2022), significant challenges persist, which could be mitigated through the adoption of modern phenotyping approaches that can potentially accelerate selection and enhance breeding efficiency.

High-throughput phenotyping (HTP) offers a transformative approach to overcome the limitations of conventional phenotyping methods in plant breeding. HTP platforms utilize advanced technologies, such as drones, ground-based sensors, and imaging systems, to rapidly and nondestructively collect high-resolution data on a wide range of plant traits, including growth, biomass, water use efficiency, and disease resistance (Shakoor et al., 2017; Wallace et al., 2018). By integrating these technologies with sophisticated data analysis techniques, HTP enables the collection of high-resolution, time-series data at unprecedented scales, providing a more comprehensive and dynamic understanding of plant performance under diverse conditions (Sheikh et al., 2024).

Integrating HTP platforms into breeding programs offers several key advantages, such as the evaluation of a larger number of individuals per unit of time and faster and repeated phenotyping, and potentially accelerating the breeding cycle (Persa et al., 2021). HTP also allows for accurate quantification of complex traits that are difficult to measure using traditional methods, such as water use efficiency, drought tolerance, and disease resistance. For instance, studies in wheat (Hassan et al., 2019; Holman et al., 2016; Schirrmann et al., 2016), maize (Dobosz et al., 2023; Liu et al., 2020), and grape (García-Fernández et al., 2021; Matese and Di Gennaro, 2018; Sepúlveda-Reyes et al., 2016) have demonstrated strong correlations between digital trits derived from HTP platforms and their ground-truth counterparts, highlighting the improved accuracy and precision of HTP-based phenotyping data.

Despite the many advantages of digital phenotyping, several critical factors must be addressed to ensure its effective implementation in breeding programs. These include the establishment of adequate field and experimental designs, standardized protocols for data acquisition and processing, and the development of scalable and sustainable operational frameworks (Lassoued et al., 2021; Ninomiya, 2022). Digital phenotyping methods enable the assessment of traits with higher heritability by capturing fine-scale morphological, physiological, and temporal traits that may not be easily measurable through traditional field-based approaches. Ultimately, for breeders to adopt and rely on digital phenotyping, the methods must be optimized to obtain consistent and accurate measurements across all stages of the breeding cycle. Remote sensing phenotyping of *M. maximus* has previously focused on biomass estimation using density factors (Sinde-González et al., 2021) and deep learning for traits, such as regrowth density and speed and dry matter yield (Santos et al., 2022; De Oliveira et al., 2021). However, reliable and scalable digital phenotyping systems that can replace conventional field measurements in *M. maximus* breeding programs have not yet been established. The systematic validation of computationally efficient digital traits, such as vegetation indices (e.g., VARI, TGI, and ExR) and texture descriptors (e.g., GLCM-derived metrics), as operational replacements for labor-intensive traditional phenotyping represents an essential step toward modernizing forage breeding. These digital proxies must demonstrate not only strong heritability and genetic correlations with target traits, such as biomass yield and forage quality, but also the practical capacity to maintain selection efficiency while reducing phenotyping costs and time requirements in breeding programs.

This study aims to advance the use of HTP and machine learning (ML) techniques in the breeding of *Megathyrsus maximus* by leveraging UAV-acquired RGB imagery. To optimize key digital phenotyping parameters, including ground sampling distance (GSD), sampling date, and effects modeling, we extracted vegetation indices and texture descriptors and evaluated their correspondence with conventionally measured agronomic traits of importance. Furthermore, ML models were applied to assess whether multivariate digital phenotypic features could improve the accuracy of trait prediction. By integrating remote sensing, digital image analysis, and statistical modeling, this research presents a comprehensive framework to support the identification and selection of superior genotypes in *M. maximus* breeding programs. Ultimately, the findings contribute to the development of more sustainable forage production systems and accelerate genetic gains through more efficient selection strategies.

## 2 Materials and methods

### 2.1 Plant material

**Figure 1:**
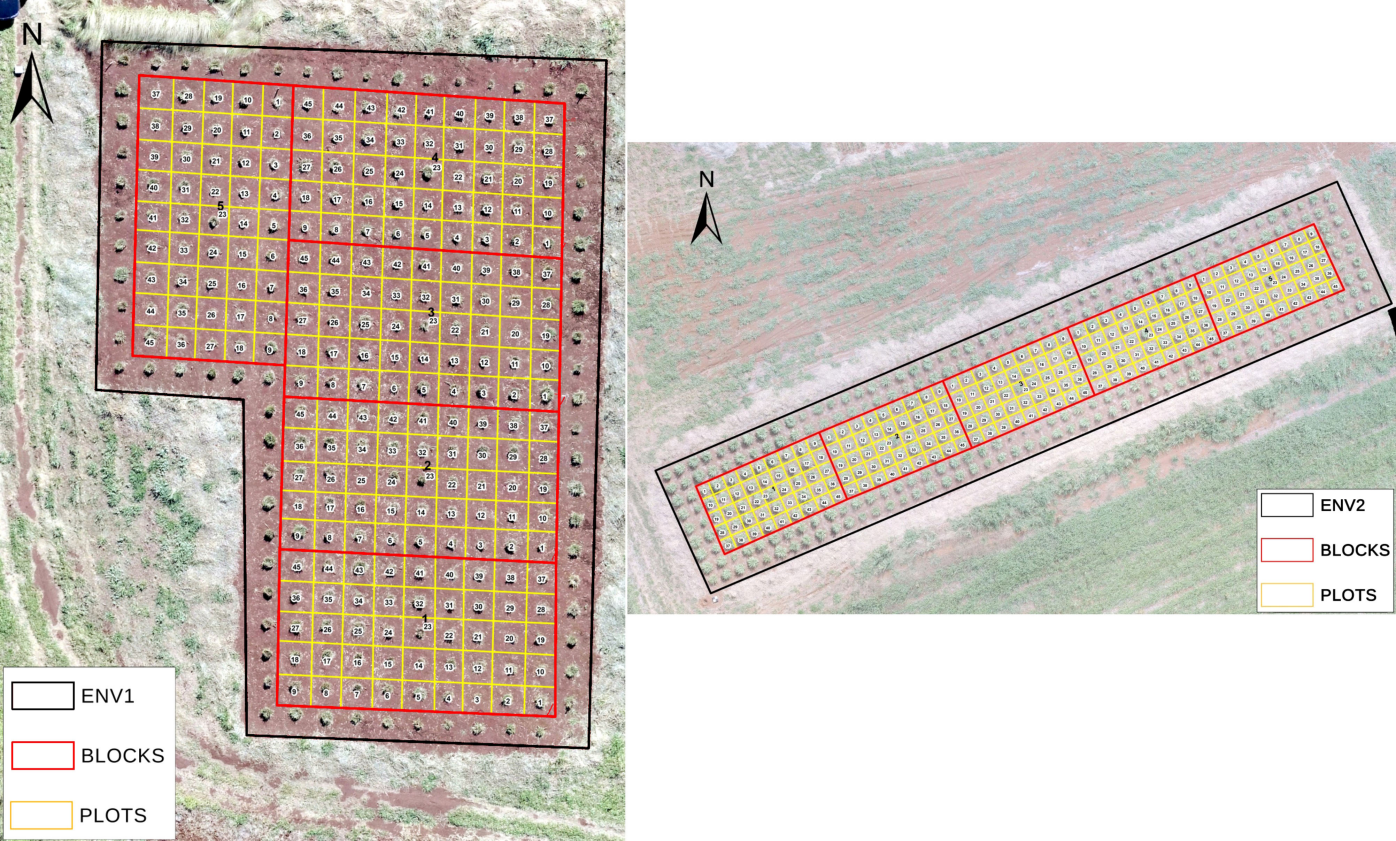
Aerial view of the two experimental sites: ENV1 (left) and ENV2 (right). Within each environment, the experimental blocks are outlined in red and individual plots in yellow.

The field experiment was conducted at the Brazilian Agricultural Research Corporation (Embrapa) Gado de Corte, located in Campo Grande, Mato Grosso do Sul, Brazil (20°27’S, 54°37’W, 530 m). A biparental population of *Megathyrsus maximus*, derived from a cross between the S12 and Miyagui cultivars, was evaluated at two distinct sites, ENV1 and ENV2 (**Figure 1**). These sites differed substantially in soil clay content (approximately 55% in ENV1 and 35% in ENV2), and were therefore treated as separate environments. This population was established on November 29, 2018, using an augmented block design with five blocks per site. Each block consisted of 45 genotypes, including three common checks-the parental lines (S12 and Miyagui) and a commercial genotype (Mombac¸a)-and 42 additional genotypes, for a total of 225 plants per field site. Phenotypic data were collected across eight assessment dates in both environments from 2019 to 2020. Canopy height (CH, cm) was measured pre-harvest as the distance from the soil surface to the mean canopy height. For harvest, plant material was cut 0.2 m from the soil surface. The fresh biomass (GMY) was immediately weighed in the field using a dynamometer (kg/plant). For dry matter analysis, the harvested material was separated into leaf, stem/sheath, and dead components, then oven-dried at 60°C to a constant weight to obtain the component dry mass (LDMY, SDMY, DMP) in g/plant. Total dry matter yield (TDMY, kg plant^−1^) was calculated as the sum of LDMY, SDMY, and DMP.

### 2.2 Image acquisition

**Figure 2:**
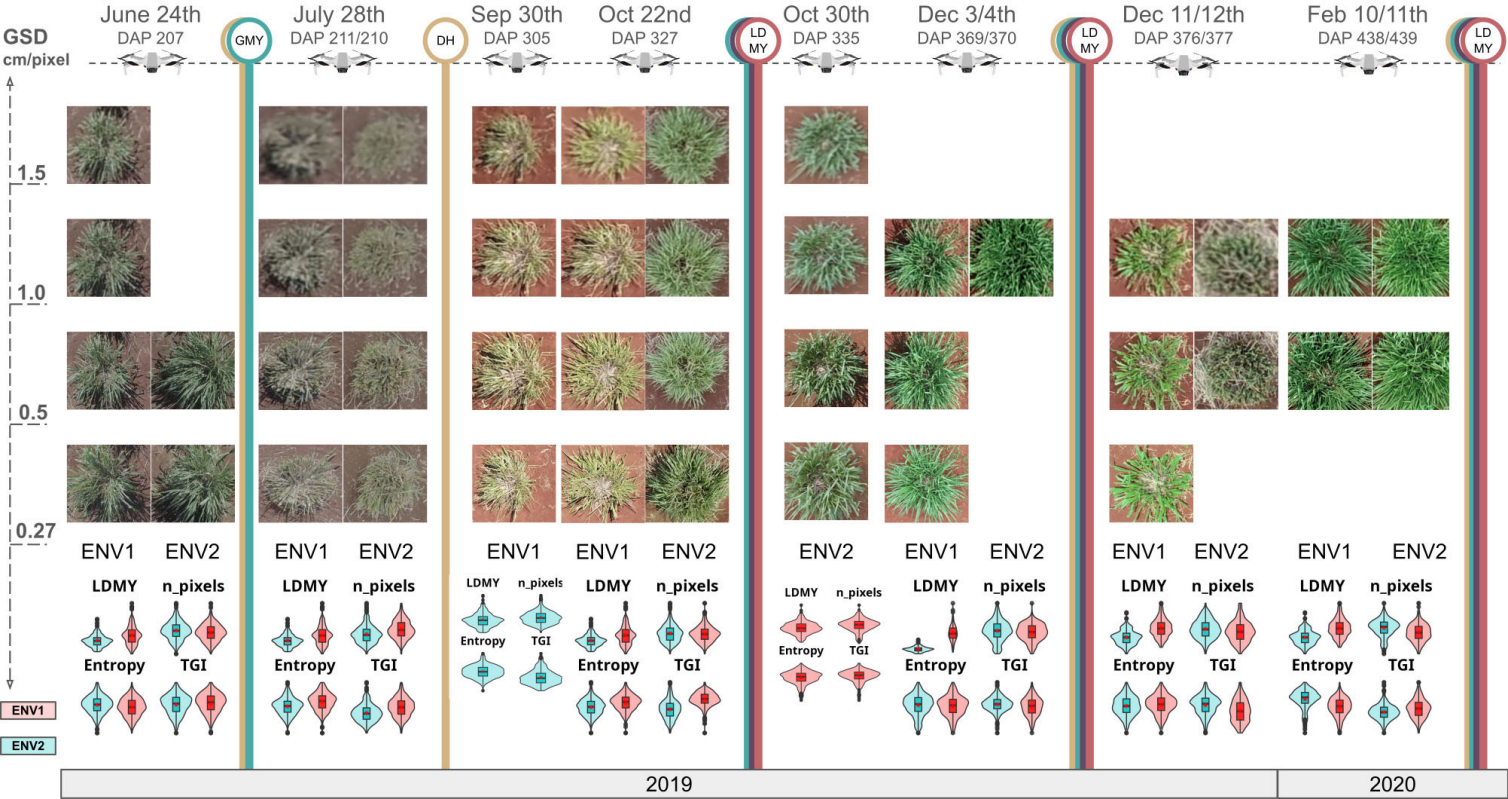
Schematic of remote-sensing imagery acquisition for two experimental environments of *M. maximus*, captured across eight dates with four ground sample distances (GSDs). Vertical stripes represent conventional traits measurement dates in correspondence to image acquisition dates (drones icon): i) green: green matter yield (GMY), ii) yellow: canopy height (CH), iii) red: leaf dry matter yield (LDMY) and iv) black: total dry matter yield (TDMY). Sample images of the Mombaça genotype are shown for each date and GSD combination. The lower section presents LDMY values for Mombaça genotype at the corresponding date and experimental environment and violin plots showcasing phenotypic data (conventional and digital) distributions for each environment across all dates.

The field images were acquired using a Phantom 4 PRO drone equipped with a high-resolution RGB sensor capable of capturing images at 5472 x 3648 pixels. Flights were conducted on dates that coincided with traditional phenotypic assessments and between 3 and 7 days after harvest. These dates are hereafter referred to as days after planting (DAP) (**Supplementary Table S1**). Image acquisition was performed with 80% frontal and lateral overlap. This resulted in repeated sampling of both environments at varying flight heights (10, 18, 35, and 55 m), yielding ground sampling distances (GSD) of 0.27, 0.5, 1.0, and 1.5 cm per pixel, respectively (**Figure 2**).

### 2.3 Image processing

For each experimental environment, an orthomosaic image was generated for each flight date and spatial resolution (GSD) using OpenDroneMap software (v3.2.1). Next, the orthomosaics were imported into QGIS (QGIS Development Team, 2025) to construct individual genotype plots. To segment the background and isolate only the plant pixels for subsequent analysis, we first computed the modified excess of green vegetation index (MExG; **Supplementary Table S1**) which enabled the binarization of the image by contrasting plant and nonplant pixels.

**Figure 3:**
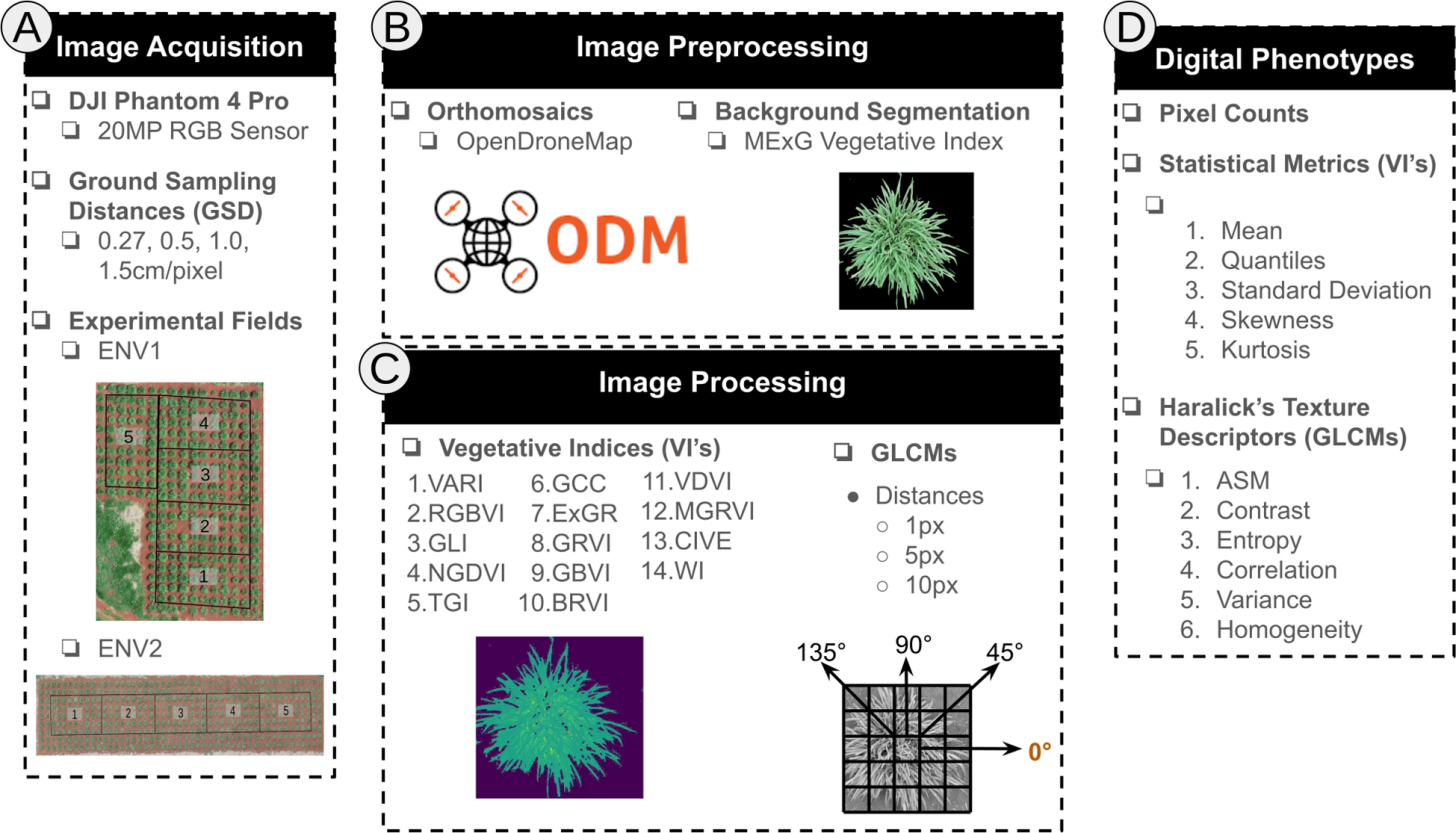
“Four stages of the digital phenotyping workflow. **(A)** Image Acquisition: Images were captured from each experimental field using a DJI Phantom 4 Pro Drone, equipped with an RGB sensor, at four different flight heights. **(B)** Image preprocessing: An orthomosaic was constructed for each drone flight using OpenDroneMap. Subsequently, non-plant pixels were removed using MExG thresholding in QGIS. **(C)** Image processing: Fourteen Vegetative Index (VIs) and twelve Gray Level Co-occurrence Matrix (GLCMs, 3 distances x 4 angles) were determined pixelwise for each genotype plot. **(D)** Digital Phenotyping: Finally, the VIs and GLCMs for each genotype plot were summarized using statistical metrics and Haralick’s texture descriptors, respectively.

Next, for each genotype and using the segmented image, the plant pixels were counted as a digital representation of the biomass (n pixels). Additionally, 14 commonly used vegetative indices (VIs) (**Supplementary Table S1**) were calculated using the image’s RGB matrix and the NumPy (v2.0) library in Python (v3.12). For each VI, seven statistical measures were computed (**Figure 3**): (i) mean, (ii) 25th percentile, (iii) 50th percentile, (iv) 75th percentile, (v) standard deviation, (vi) skewness, and (vii) kurtosis. To characterize spatial patterns within the segmented plant images (**Figure 3**), twelve gray-level co-occurrence matrices (GLCMs) were generated using the scikit-image (v0.24) Python package. These matrices were constructed using three distance values (1, 5, and 10 pixels) and four angles (0°, 45°, 90°, and 135°) to capture textural variations at different spatial scales and orientations. From each GLCM, six texture descriptors were extracted: angular second moment (ASM), correlation, entropy, contrast, homogeneity, and variance (**Supplementary Table S1**). Finally, all the digital features were normalized using min-max scaling, which transforms each feature to a fixed range of [0,1] (Equation 1), where x is the original feature value, min(x) is the minimum value in the feature, max(x) is the maximum value in the feature, and x’ is the normalized value.

(1) $$x^{\prime }=\frac{x-\min\;(x)}{\max\;(x)-\min\;(x)}$$

A total of 44 high-quality orthomosaics were generated from the acquired aerial imagery, 25 for ENV1 and 19 for ENV2 (**Supplementary Figure S1**, **S2** respectively). For each mapped genotype plot in QGIS, 171 digital traits were computed, encompassing a range of image-based traits. This dataset included 7 statistical metrics (mean, quantiles, standard deviation, skewness, and kurtosis) calculated from 14 vegetative indices (98 digital traits), five texture descriptors (ASM, entropy, variance, homogeneity, contrast, and correlation) calculated from 12 gray-level co-occurrence matrices (GLCMs) (72 digital traits) using multiple angle (0°, 45°, 90°, and 135°) and pixel distance (1, 5, and 10 pixels) combinations, and a single plant pixel count measure (1 digital phenotype).

To address the high dimensionality of the dataset, a collinearity analysis was performed separately for each type of descriptor to identify and remove redundant features. For vegetative indices, Pearson’s correlation was calculated between their statistical measures, whereas for texture descriptors, correlation was assessed between different angle and distance combinations. In both cases, descriptor pairs with a Pearson correlation coefficient greater than 0.75 were considered collinear, and one feature from each highly correlated pair was removed to eliminate redundancy while preserving the maximum amount of information. To investigate the relationships between image-derived traits and conventionally measured phenotypic values, pairwise Pearson’s correlation coefficients (r) (Equation 2) were calculated using normalized data, where r is the Pearson correlation coefficient, *x_i_*represents the individual values of the image-derived trait, $y_{i}$ represents the individual values of the conventionally measured phenotypic trait, $\bar{x}$ is the mean of the image-derived trait values, and $\bar{y}$ is the mean of the conventionally measured phenotypic 149 trait values:

(2) $$r=\frac{\sum (x_{i}-\bar{x})\left(y_{i}-\bar{y}\right)}{\sqrt{\sum {\left(x_{i}-\bar{x}\right)}^{2}\sum {\left(y_{i}-\bar{y}\right)}^{2}}}$$

The calculation of the Pearson correlation coefficients was followed by a t test (Equation 3) to determine the coefficients’ statistical significance, where t is the t statistic for testing the significance of the correlation coefficient, r is the Pearson correlation coefficient and n is the number of observations:

(3) $$t=\frac{r\sqrt{\;n-2\;}}{\sqrt{\;1-r^{2}\;}}$$

Image data, acquired across multiple flight heights (GSDs) within a single imaging date, were matched to corresponding conventional measurements (**Supplementary Table S2**) for that date to ensure that comparisons were made between physiologically equivalent stages, thus mitigating potential discrepancies arising from the multiple GSDs. Images acquired 3 to 7 days after the harvest were matched to the next conventional measurement date available to evaluate whether future values could be predicted from an early regrowth stage.

### 2.4 Prediction system

Four machine learning (ML) regression algorithms were evaluated for their ability to predict phenotypic traits when trained on the generated digital traits using Python v3.12 and the scikit-learn library (v1.4.2) (Pedregosa et al., 2011): (a) adaptative boosting (AdaBoost) (Freund and Schapire, 1997), (b) multilayer perceptron (MLP) neural networks (Popescu et al., 2009), (c) random forests (Breiman, 2001) (RF), and (d) support vector machines (SVMs) (Cristianini and Shawe-Taylor, 2000). All algorithms were trained using default scikit-learn hyperparameters, without tuning, to ensure computational feasibility and consistent comparability across the large number of model combinations evaluated. For AdaBoost, the base estimator was a decision tree regressor with a linear loss function for weighting boosting interactions. The MLP neural network was constructed with a single hidden layer comprising 100 neurons activated by the rectified linear unit (ReLU) function (Equation 4), where r is the input to the neuron:

(4) $$\text{ReLU}\left(x\right)=\{\begin{array}{ll} x, & x\geq 0\\ 0, & x\leq 0 \end{array}$$

For the RF model, the forest was composed of 100 trees, and the mean squared error (MSE) was used as the criterion for evaluating the quality of the splits. Each internal node required a minimum of 2 samples to initiate a split, while the leaf nodes required at least 1 sample. The trees were allowed to grow without any maximum depth, and bootstrapping was employed during training. SVM regression was implemented using the default radial basis function (RBF) kernel from scikit-learn. The RBF kernel is defined as follows (Equation 5):

(5) $$\text{RBF}\left(x_{i},x_{j}\right)=\text{exp }\left(-\frac{d{\left(x_{i},x_{j}\right)}^{2}}{2l^{2}}\right)$$

(6) $$\text{RMSE}=\sqrt{\frac{1}{n}\sum_{i=1}^{n}{\left(y_{i}-{\hat{y}}_{i}\right)}^{2}}$$

(7) $$R^{2}=1-\frac{\sum_{i=1}^{n}{\left(y_{i}-{\hat{y}}_{i}\right)}^{2}}{\sum_{i=1}^{n}{\left(y_{i}-\bar{y}\right)}^{2}}$$

In these equations, *n* represents the number of samples, ${\hat{y}}_{i}$ represents the predicted conventional phenotypic value, $y_{i}$ represents the corresponding ground-truth value, and $\bar{y}$ represents the mean of the corresponding ground-truth values.

These models predicted the conventional plant traits on the basis of the calculated digital traits (**Figure 3**) for each combination of location, image acquisition date and GSD, hereafter referred to as a flight. Model training and evaluation were conducted independently for each flight using repeated 4-fold cross-validation with 100 repetitions (400 train–validation splits) implemented via the *RepeatedKFold* class in scikit-learn. In each split, the model was trained on the training folds and evaluated on the held-out validation fold. Performance metrics—root mean squared error (RMSE, Equation 6), coefficient of determination (*R*^2^, Equation 7), and Pearson’s correlation coefficient (*r*)—were computed from the out-of-fold predictions and averaged across all 400 splits.

To assess differences in prediction accuracy, we performed ANOVA followed by the Scott-Knott multiple comparison test (Jelihovschi et al., 2014) and Pearson’s correlation with the validation dataset using R (Team, R. C, 2025). Additionally, we evaluated differences in model prediction errors (RMSEs) for the flights that could be accurately predicted (r *>* 0.75). For these flights, we calculated the mean RMSE across cross-fold validations obtained in the training step and considered the difference significant if any model for the same flight showed a difference in the mean RMSE value greater than 0.1 compared with the others.

Feature importance was extracted from the trained RF regressor, where higher scores indicate greater contribution to prediction accuracy. A feature selection threshold was visually determined from a line plot of importance scores grouped by flight, corresponding to the natural inflection point between the dense cluster of near-zero scores and the discrete peaks of dominant features. This process ensured that only the most predictive image features were retained for further analysis.

### 2.5 Mixed-effects modeling

Statistical analysis of the phenotypic data was performed using linear mixed-effects models in the R environment (Team, R. C, 2025) with the ASReml-R package (The VSNi Team, 2023). Significance testing for fixed and random effects was conducted via Wald F tests and likelihood ratio tests (LRTs) within analyses of deviance (ANODEV), respectively. Variance components were estimated using Restricted Maximum Likelihood (REML). Subsequently, Best Linear Unbiased Predictors (BLUPs) and Best Linear Unbiased Estimates (BLUEs) (de Resende, 2000) were derived for the progenies and checks, respectively, to predict the breeding values (BVs). Finally, Pearson’s correlation and Principal Component Analysis (PCA) were performed on predicted BVs.

The following model was applied to obtain adjusted conventional phenotypic values within each environment (Equation 8):

(8) $$y_{ijkmn}=\mu +C_{n/m}+H_{j}+CH_{nj}+G_{i/m}+GH_{ij}+B_{jk}+\epsilon_{ijk}$$

Where $y_{ijkmn}$ is the phenotypic value of the i-th genotype in the j-th harvest within the k-th block; μ is the overall population mean. The genetics effect were divided in two groups, $G_{i/m}$ and $C_{n/m}$, representing respectively the random effect of the i-th progeny line ( $G_{i/m}∼N\left(0,\sigma_{G}^{2}\right)$), with $\sigma_{G}^{2}$ representing the genetic variance) and the fixed effect of the n-th checks (control) both within the m-th group. Similarly, the effects of the genotype by harvest interaction were also separated in $GH_{ij}$ ( $GH_{ij}∼N\left(0,\sigma_{GH}^{2}\right)$, with $\sigma_{GH}^{2}$ representing the genetic variance) and $CH_{nj}$. $H_{j}$ is the fixed effect of the harvest; $B_{jk}∼N\left(0,\sigma_{B}^{2}\right)$ is the random effect of the k-th block within the j-th harvest and associated variance $\sigma_{B}^{2}$; and $\epsilon_{ijk}$ is the random effect of the residual errors ( $\epsilon_{ijk}∼N\left(0,\sigma_{\epsilon }^{2}\right)$), with $\sigma_{\epsilon }^{2}$ being the error variance. All random effects were considered to be independent and identically distributed.

To account for environmental and trait-specific effects, digital traits were analyzed separately for each environmental and trait combination using the following mixed-effects model (Equation 9):

(9) $$y_{ijkmn}=\mu +C_{n/m}+H_{k}+G_{i/m}+B_{j}+\epsilon_{ijk}$$

Where $y_{ijkmn}$ is the phenotypic value of the i-th genotype in the j-th block within the k-th GSD; μ is the overall population mean. The genetics effect were divided in two groups, $G_{i/m}$ ( $G_{i/m}∼N\left(0,\sigma_{G}^{2}\right)$), with $\sigma_{G}^{2}$ representing the genetic variance) and $C_{n/m}$, representing respectively the random effect of the i-th progeny lines and the fixed effect of the n-th checks (control) both within the m-th group; $H_{k}$ is the fixed effect of the GSD; $B_{j}∼N\left(0,\sigma_{B}^{2}\right)$ is the random effect of the j-th block; and $\epsilon_{ijk}$ is the random effect of the residual errors, ( $\epsilon_{ijk}∼N\left(0,\sigma_{\epsilon }^{2}\right)$), with $\sigma_{\epsilon }^{2}$ being the error variance. All random effects were considered to be independent and identically distributed.

To estimate the proportion of genetic influence on both conventional and digital traits, broad-sense heritability ( $H^{2}$) and Cullis heritability ( $H_{C}^{2}$) were calculated for each trait using Equation 10 and Equation 11, respectively:

(10) $$H^{2}=\frac{\sigma_{G}^{2}}{\sigma_{P}^{2}}$$

(11) $$H_{C}^{2}=1-\frac{PEV}{2\sigma_{G}^{2}}$$

where *PEV* denotes the prediction error variance (average variance of individual plant comparisons), $\sigma_{G}^{2}$ represents the genetic variance, and $\sigma_{P}^{2}$ represents the phenotypic variance.

Following mixed-effects modeling and BV estimation for both conventional and digital traits, we employed a coincidence index analysis to quantify whether digital phenotyping identifies the same superior genotypes as conventional methods do. For each trait, genotypes were ranked by BV values, and selection thresholds were established at three intensities (top 5%, 20%, and 50%). Coincidence indices (CIs, Equation 12) were calculated between trait pairs as the percentage of common genotypes in both selections.

(12) $$CI=\frac{\text{number of common genotypes}}{\text{total genotypes in the percentile}}\times 100$$

This approach quantifies whether digital phenotyping identifies the same superior genotypes as conventional methods do, validating its potential utility in breeding programs and providing breeders with candidates that combine desirable characteristics across multiple traits.

## 3 Results

### 3.1 Image processing

To address multicollinearity, we performed a correlation analysis among the statistical metrics used to summarize the vegetation indices. This analysis revealed strong linear relationships (*r >* 0.75) among the quantiles, mean, and standard deviation for nearly every index (**Supplementary Figure S3**). As a result, the 50th and 75th percentiles were removed because of their consistently high correlations. Similarly, we found that different combinations of distance and angle for calculating gray-level co-occurrence matrices (GLCMs) produced highly correlated features (*r >* 0.95) (**Supplementary Figure S4**). To reduce data dimensionality and redundancy, a distance of 0 and an angle of 10° were selected for all subsequent GLCM calculations. Ultimately, from an initial set of 171 computed digital traits (98 from VIs, 72 from GLCMs, and the pixel count), a reduced set of 76 features (70 from VIs, 5 from GLCMs, and the pixel count) was used for subsequent analyses.

### 3.2 Pairwise correlations between digital and conventional traits

**Figure 4:**
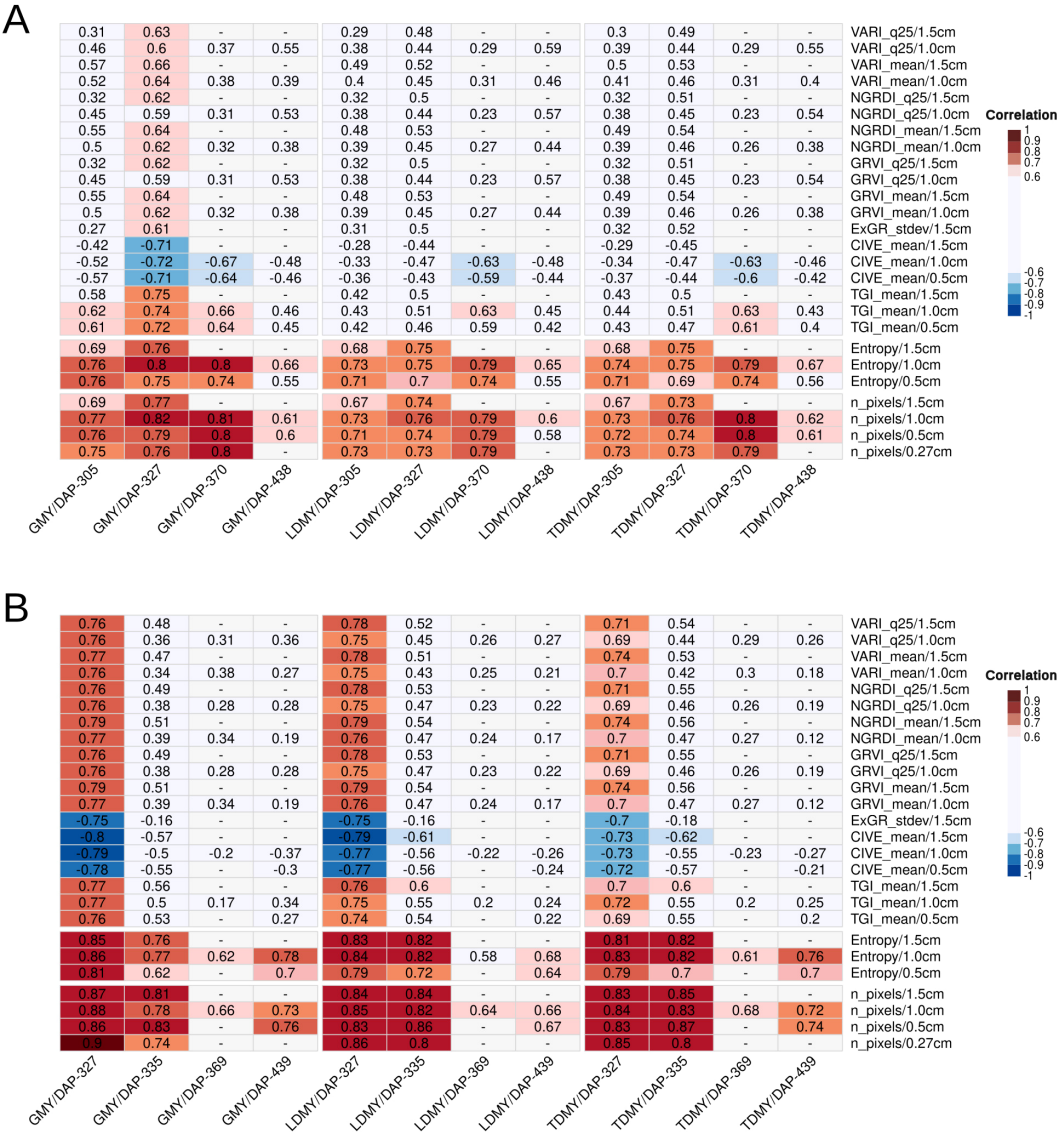
Heatmap of Pearson correlation coefficients (*r*) between digital traits extracted from images at four ground sample distances (GSDs) and conventional traits, measured across eight sampling dates and two field locations: **(a)** ENV1 and **(b)** ENV2. The y-axis lists digital trait-GSD combinations; the x-axis shows conventional traits grouped by DAP. Digital trait-GSD combinations are included only if they exhibit strong correlations (*r >* 0.75) with any conventional trait on at least one date or location.

Pearson’s correlation analysis revealed both consistency and environment-specific variations in the relationships between digital traits and their conventional counterparts across the two experimental fields. Strong correlations (*r >* 0.75) were consistently observed between the digital traits and all conventional biomass yield traits (GMY, LDMY, and TDMY) at both locations and across GSDs. demonstrating the robustness of digital yield estimation methods. In contrast, the canopy height (CH) exhibited only moderate correlations, with maximum coefficient values of 0.60 (with GCC stdev in ENV1) and 0.61 (with WI stdev in ENV2) (**Supplementary Table S3**). This moderate performance was expected, as remotely sensed aerial imagery provides only two-dimensional scene representation and cannot detect plant components beneath the canopy cover without derived digital surface and terrain models. At ENV1, significant correlations between digital and yield traits were observed across three key assessment dates: 305, 327, and 370 days after planting (DAP) (**Figure 4A**).

The strongest correlations occurred at DAP-370, where most coefficients for Entropy and n pixels ranged from 0.79 to 0.82 at a ground sample distance (GSD) of 1.0 cm/pixel. Among the vegetation indices (VIs), only TGI mean was strongly correlated with GMY at this location, achieving peak performance on DAP-327 at 1.5 cm/pixel GSD.

Compared with ENV1, ENV2 consistently demonstrated substantially stronger correlations between digital and yield traits parameters (**Figure 4B**). For entropy and n pixels, the correlation coefficients in ENV2 ranged from 0.76 to 0.90, with most of them being greater than 0.8 across multiple assessment dates (DAP-327, DAP-335, DAP-369 and DAP-439).

Specifically, six vegetation indices-VARI (q25 and mean), NGRDI (q25 and mean), GRVI (q25 ad mean), ExGR (stdev), CIVE (mean), and TGI (mean)-showed strong correlations (r ranging from 0.75 to 0.80) with yield traits, exclusively on DAP-327 and with TDMY. This particular growth stage also exhibited the highest correlations with image features, notably n pixels (*r* = 0.90) and entropy (*r* = 0.86). On DAP-439, the final phenotyping date, when plants typically reached peak vigor, strong correlations for both GMY and TDMY were observed in ENV2 but not in ENV1. This suggests that digital phenotyping maintained its predictive power even at advanced growth stages in ENV2. However, LDMY did not exhibit strong correlations with any digital traits in either environment on this date, with peak coefficients reaching only *r* = 0.68, indicating that dry matter estimation is less accurate on this phenotyping date. Interestingly, DAP-335 represents one of the dates when image acquisition was performed just a few days after harvest, when the plants had recently regrown. The digital traits derived from these images were used to predict conventional phenotypic values that were measured only one month later (DAP-369), demonstrating the predictive potential of early postharvest imaging for traits assessed at later growth stages. Remarkably, the correlations achieved with the DAP-335 imagery were even greater than those obtained from derived traits using images taken one day before the DAP-369 harvest, although this comparison is limited because the DAP-369 images for ENV2 are available only for a single GSD (1.0 cm/pixel).

The results demonstrate a significant environmental influence on digital phenotyping efficacy for yield prediction, with ENV2 consistently outperforming ENV1. DAP-327 emerged as particularly important, representing the peak performance timing for ENV2 while also demonstrating strong correlations in ENV1, suggesting that this growth stage may offer optimal conditions for digital phenotyping across diverse environments. Environment-specific temporal patterns were evident-DAP-370 produced the strongest correlations (*r >* 0.8) in ENV1 but only moderate correlations (0.6< *r<* 0.68) in ENV2. Conversely, DAP-439 demonstrated strong correlations for GMY and TDMY in ENV2 but showed only moderate performance in ENV1 (0.55< *r<* 0.67).

Direct environmental comparisons were constrained by flight schedule limitations. DAP-305 data were available exclusively in ENV1, whereas DAP-335 flights occurred only in ENV2. Additionally, DAP-370, which exhibited peak correlations in ENV1 (*r* = 0.85), had limited data availability in ENV2 because of technical constraints that restricted analysis to single GSD measurements. With respect to spatial resolution effects, the 0.5 and 1.0 cm/pixel GSDs performed comparably on dates when all the resolutions were available. The highest resolution (0.27 cm/pixel) was significantly correlated only with n pixels at ENV2 on DAP-327 for GMY prediction. Notably, this represented the strongest correlation (r = 0.90) observed across all environments, assessment dates, and traits. The variable performance of digital traits across environments and assessment dates highlights the complexity of genotype-environment interactions in digital phenotyping applications. Vegetation indices demonstrated environment-dependent efficacy, performing effectively in ENV2 but showing limited utility in ENV1, while structural traits, such as pixel counting and entropy, maintained consistent predictive values across both environments. Other Haralick’s texture features, including the angular second moment (ASM), homogeneity, and variance, occasionally exhibited moderate negative correlations (*r<* −0.70) but failed to achieve consistent significance thresholds. Among statistical summarization methods, measures of central tendency (mean and 25th percentile) and dispersion (standard deviation) consistently outperformed higher-order statistical moments, such as skewness and kurtosis, across all digital trait categories, with the latter showing negligible correlations with yield parameters. These findings also suggest that machine learning approaches capable of integrating information from multiple digital trait categories and assessment dates may overcome the limitations of individual correlation-based predictors and improve overall yield prediction accuracy under diverse environmental conditions.

### 3.3 Predicting conventional traits using ML algorithms

Machine learning algorithms were employed to evaluate whether combining multiple digital traits could improve the accuracy of phenotype prediction beyond individual trait correlations, particularly for the assessment of flight dates and traits (specifically CH), for which nonsignificant correlation coefficients were detected. Across the twenty-five distinct flight groups (DAP + GSD) in ENV1, ten demonstrated strong predictive accuracy (**Supplementary Figure S5**). Specifically, 45 of the 316 evaluated prediction groups (location × DAP × GSD × trait × model combinations) yielded *r >* 0.75 with ground truth data (**Supplementary Table S4**). Five of the evaluated flight dates (DAP-207, DAP-305, DAP-327, DAP-370, and DAP-438) were significantly correlated with at least one of the conventional traits (**Table 1**), and a marked improvement over the three dates (DAP-305, DAP-237, and DAP-370) was identified through pairwise correlation. Notably, DAP-438 emerged as a critically important newly accurate prediction date. This flight date, performed at the close of the phenotyping period during the wet season, captures genotypes at their peak vigor and production. Such data are invaluable for breeders, as they reflect optimal genotype performance, making accurate remote sensing phenotyping on this date highly desirable for breeding programs.

**Table 1:** Pearson’s correlation coefficients between ML-based predicted values of conventional traits with corresponding ground truth data.

DAP	207		211/210		305	327		335	370/369		377/376		438/439	
ENV	1	2	1	2	1	1	2	2	1	2	1	2	1	2
CH	0.63	0.50	0.59	0.65	0.74	0.34	0.45	0.47	0.51	0.46	0.48	0.41	0.71	0.59
GMY	**0.76**	**0.74**	0.50	0.66	**0.76**	**0.81**	**0.90**	**0.82**	**0.86**	0.73	0.58	0.45	0.70	**0.81**
LDMY	0.72	0.71	0.49	0.59	0.73	0.73	**0.88**	**0.83**	**0.81**	0.64	0.63	0.41	**0.78**	**0.77**
TDMY	0.72	0.70	0.50	0.58	0.73	0.73	**0.82**	**0.84**	**0.81**	0.68	0.64	0.39	**0.78**	0.70

DAP, days after planting; CH, canopy height; ENV, environment; GMY, green matter yield; LDMY, leaf dry matter yield; TDMY, total dry matter yield. Bold: values ≥ 0.75; 0.74 included as near-threshold.

An improvement in the prediction of canopy height (CH) was also observed. In ENV1, CH showed no significant pairwise correlation with any individual digital trait across all the dates, with a maximum correlation coefficient of only 0.61 (4B). When machine learning was used, however, the prediction accuracy increased, reaching a correlation of 0.71 with the MLP model at DAP-438, which was still below the significance threshold but notably closer. All yield-related traits (GMY, LDMY, and TDMY) could be predicted with high accuracy (*r >* 0.8; **Table 1**) at least one DAP.

**Figure 5:**
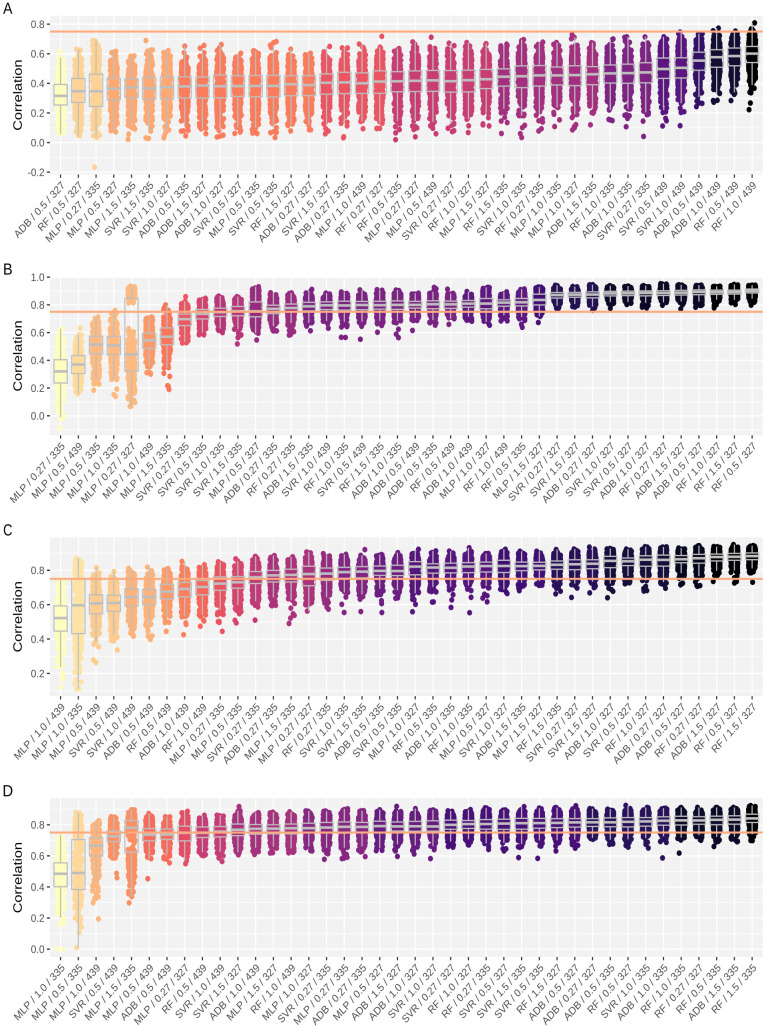
Performance comparison of machine learning algorithms for predicting conventional traits from remote sensing data in ENV2. Digital traits were acquired with varying combinations of dates (DAP) and ground sample distance (GSD). Only DAP showing a significant mean Pearson correlation with ground truth data for at least one conventional trait are included. **(A)** Canopy height (CH), **(B)** green matter yield (GMY), **(C)** leaf dry matter yield (LDMY), and **(D)** total dry matter yield (TDMY). Algorithms evaluated include multilayer perceptron (MLP), random forest (RF), support vector regression (SVR), and adaptive boosting (AdaBoost).

In ENV2, ten of the nineteen flight groups also showed strong predictive accuracy (**Figure 5**), accounting for 91 of the 304 prediction means evaluated-more than double the number observed in ENV1 (**Supplementary Table S4**). This increased accuracy was largely driven by the 0.27 cm/pixel and 1.5 cm/pixel GSDs, which yielded more significant predictions in ENV2 than in ENV1. However, these accurate predictions corresponded to the same significant dates (DAPs 327, 335, 369, and 439) previously identified by pairwise correlation analysis. Notably, early-season imaging at DAP-207 remained poorly correlated with digital traits, contradicting what was observed in ENV1 and indicating that environmental conditions strongly influence the agreement between remote sensing predictions and ground-based phenotyping. Canopy height (CH) prediction showed only a modest improvement over the best pairwise correlation (WI stdev, *r* = 0.61), reaching a maximum Pearson correlation of 0.65, also with the MLP, at DAP-210 (**Table 1**). Interestingly, this flight occurred three days after harvest and was used to predict future CH measurements recorded three months later (DAP-312).

Despite the moderate correlation, these results suggest that part of the variation in plant height may be explained by tussock characteristics, such as diameter, texture and color, which are observable only after harvest when leaves are removed. In contrast, yield-related traits showed consistent gains, building on already high baseline correlations. GMY, in particular, reached a mean correlation of 0.85 across the models and GSDs at DAP-327, with the highest correlation (*r* = 0.90) achieved using a random forest (RF) model at 0.5 cm/pixel resolution, followed by LDMY on the same date using the same model (*r* = 0.88, GSD = 1.5 cm/pixel) and TDMY on DAP-335 with the RF model (*r* = 335, GSD = 1.5 cm/pixel).

As depicted by the color coding in **Figure 5** (ENV2) and **Supplementary Figure S5** (ENV1), Tukey’s HSD test-based grouping highlights that prediction accuracy is deeply connected with the date of image acquisition and that predictions on the same date are frequently grouped together independently of the model algorithm or image resolution (GSD). When the performance of the machine learning algorithms was compared, RF consistently demonstrated the greatest number of significant predictions across both environments. In ENV1, the RF model accurately predicted 17 different flight groups, followed by the ADB (15), SVR (8), and MLP (5) models. This trend continued in ENV2, where the RF model again led with 28 significant prediction means, closely followed by the ADB (27), SVR (24), and MLP (12) models.

Notably, the MLP model consistently produced the fewest significant predictions overall, which is likely attributable to the use of default model parameters without extensive hyperparameter tuning. However, it is crucial to highlight that the MLP model achieved the best individual prediction for CH, underscoring its potential to extract abstract aspects from images that can be correlated with the vertical growth dynamics of the plant.

Model performance was also examined by identifying prediction groups with RMSE differences greater than 0.1 between any model pair. Among the 36 groups with substantial differences, ENV1 accounted for 11 cases (**Supplementary Figure S6**), while ENV2 represented the majority with 25 cases (**Supplementary Figure S7**). Regarding traits, CH showed the most differences (18 cases: 8 from ENV1 and 10 from ENV2). Large RMSE differences occurred mainly in lower-accuracy predictions, with 18 cases having *r* ≤ 0.75 and 12 cases having *r >* 0.75. Across all 316 prediction scenarios, the MLP model most frequently exhibited the highest RMSE (54 cases), followed by ADB (15 cases), SVR (9 cases), and RF (1 case). These results highlight the consistent reliability of the RF model and the greater variability observed in MLP performance.

**Figure 6:**
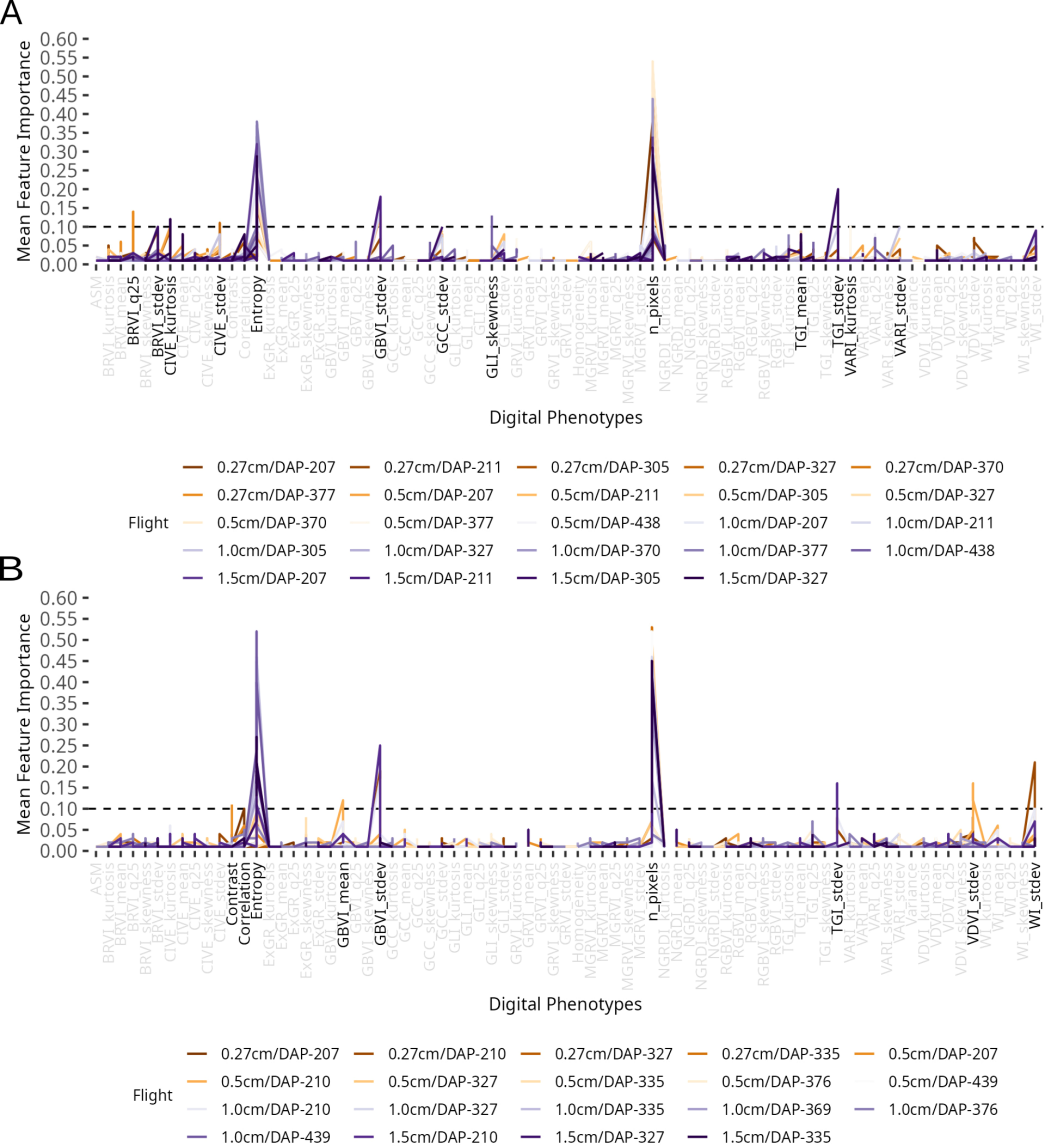
Digital traits contributions to prediction of conventional traits using the random forest algorithm. Mean importance values were determined using the scikit-learn algorithm. **(A)** ENV1. **(B)** ENV2.

To identify the digital traits most influential in accurately predicting conventional counterparts, we performed a feature importance analysis (**Figure 6**). We set a feature importance threshold of 0.1, as it visually separates the most significant features from the remaining features. In ENV1, 13 digital traits were considered important (mean feature importance *>* 0.1), including seven vegetation indices (BRVI, CIVE, GBVI, GCC, GLI, TGI, and VARI), one texture descriptor (entropy), and the pixel count (n pixel) (6A). Among these, entropy and n pixel were the most influential by a wide margin, each with an importance score exceeding 0.35. In ENV2, the feature importance results showed a more complementary pattern between the image processing approaches, with key contributions from four vegetation indices (GBVI, TGI, VDVI, and WI), three texture descriptors (contrast, correlation, and entropy), and the pixel count (n pixel) (**Figure 6B**). As in ENV1, entropy and n pixel again emerged as the most important traits, with mean importance values greater than 0.35. These results suggest that compared with color-based vegetation indices, the physical characteristics of a plant-specifically its canopy complexity and overall size-are superior indicators of yield. Entropy, a measure of image texture, likely captures crucial information about a plant’s structural density and leaf overlap, which are directly related to biomass accumulation. Similarly, the pixel count serves as a direct proxy for a plant’s projected size, which is a fundamental measure of its growth and health.

### 3.4 Evaluation of phenotypes: variance components, heritability and predicted breeding values

To evaluate conventional and digital traits and quantify the genetic and environmental contributions to their observed variance, mixed-effects models were employed. For digital traits, our models incorporated data exclusively from the imaging acquisition date (DAP-327), which demonstrated the strongest correspondence with conventional traits in booth locations. Furthermore, only those digital traits surpassing the predefined importance threshold for machine learning prediction ( >0.1) in both experimental environments were included (n_pixels, entropy, TGI_stdev, GBVI_stdev and WI_stdev).

Wald’s significance tests for fixed effects revealed consistent patterns across both conventional and digital phenotyping approaches (**Table 2**). For conventional traits, the harvest date (phenotyping dates within the same cut), genotypic effects of the checks, and checks × harvest interaction were significant factors influencing all conventional traits at both locations (**Supplementary Table S5**). With respect to the digital traits, the genotype effect of the checks and ground sampling distance (GSD) effect were significant across both environments and all the digital traits evaluated. Random effects estimation revealed significant block × harvest interactions for all conventional traits in both environments (**Table 2**). The genotype effects for the progeny were consistently significant across all the conventional and digital traits in both environments. In contrast, the genotype × harvest interaction was not significant for any conventional traits in either environment. Environmental factors were also influential, with conventional traits being primarily impacted by block × harvest interactions, whereas digital traits were significantly affected by ground sampling distance (GSD).

**Table 2:** Estimated variance components, heritability, and significance of random effects for conventional and selected digital traits.

Location	Parameter	LDMY	TDMY	GMY	CH	n_pixels	Entropy	GBVI	TGI	WI
ENV1	Block	–	–	–	–	0.03***	0.02***	0.02***	0.03***	0.02***
	Block + Harvest	0***	0***	0.04***	8.81***	–	–	–	–	–
	Progeny	0.01***	0.01***	0.24***	36.51***	0***	0***	0.01***	0***	0***
	Progeny + Harvest	3.90E-10	1.20E-08	3.70E-07	0	–	–	–	–	–
	$H^{2}$	0.87	0.85	0.82	0.7	0.84	0.8	0.43	0.83	0.55
	$H_{C}^{2}$	0.94	0.93	0.91	0.87	0.99	0.98	0.95	0.99	0.96
ENV2	Block	–	–	–	–	0.02***	0.02***	0.02***	0.02	0.01***
	Block + Harvest	0.01***	0.02***	0.82***	16.1***	–	–	–	–	–
	Progeny	0.02***	0.04***	1.08***	32.94***	0.01***	0***	0***	0***	0.01***
	Progeny + Harvest	3.40E-08	5.10E-08	1.00E-06	70.21***	–	–	–	–	–
	$H^{2}$	0.78	0.81	0.79	0.43	0.74	0.76	0.51	0.82	0.47
	$H_{C}^{2}$	0.91	0.92	0.94	0.93	0.99	0.99	0.96	0.98	0.96

*H*^2^, Broad-sense heritability; ${H\;}_{C}^{2}$, Cullis heritability; CH, canopy height; DAP, days after planting; ENV, environment; GMY, green matter yield; LDMY, leaf dry matter yield; TDMY, total dry matter yield; n pixels, count of plant pixels after background removal; Entropy, Haralick texture descriptor; GBVI, green-blue vegetation index; TGI, triangular greenness index; WI, Woebbecke’s index. *, ** and ***: Significant at the 0.05, 0.01 and 0 probability level respectively.

Standard broad-sense heritability ( $H^{2}$) estimates were consistent for most traits across different locations, with the only exception being CH, whose $H^{2}$ was much higher in ENV1 (0.7) than in ENV2 (0.42) (**Table 2**). Specifically, n_pixels, entropy, and TGI had high heritability ( $H^{2}>0.75$), whereas the GBVI and WI showed moderate heritability, with values of 0.47 and 0.55 in ENV1 and ENV2, respectively. Cullis heritability ( $H_{C}^{2}$), estimated using the BLUPs values showed much greater consistency and higher values in both locations. All the evaluated traits had $H_{C}^{2}$ values greater than 0.91, even those with only moderate broad-sense heritability ( $H^{2}$). The highest heritability values were observed for the digital traits, which surpassed those for the conventional traits. The high heritability of these digital traits confirms that they have a strong genetic component, making them excellent candidates for use in forage breeding programs to increase selection efficiency.

**Figure 7:**
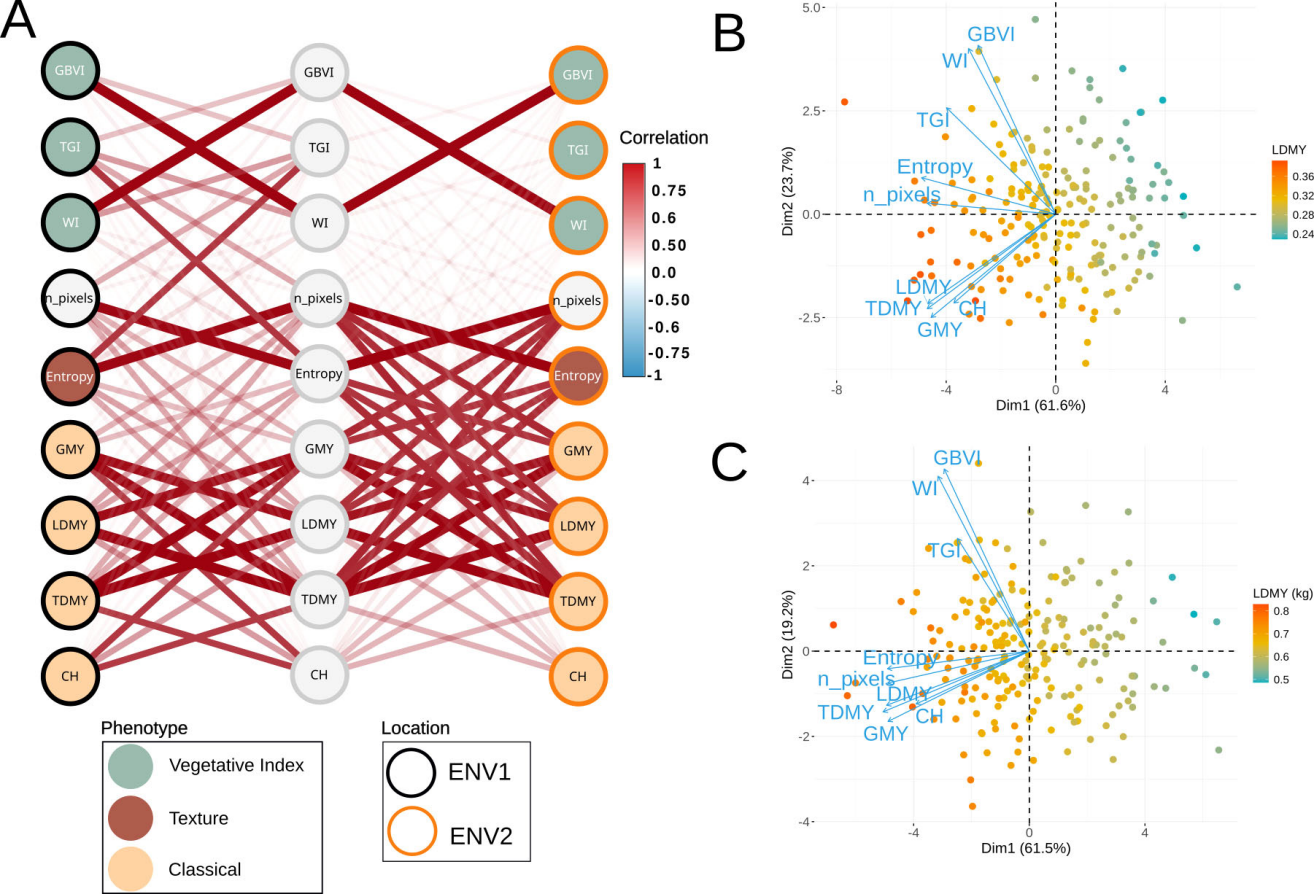
**(A)** Correlation network between predicted breeding values (BVs) of digital and conventional traits in ENV1 and ENV2. Principal component analysis (PCA) of BVs for **(B)** ENV1 and **(C)** for ENV2.

Correlations between predicted BVs were analyzed to determine whether the significant correlations observed in prior analyses would persist after experimental and environmental effects that influence phenotypic values were removed (**Figure 7A**). Using the predicted BVs, ENV1 exhibited only weak to moderate correlations between digital traits and yield traits and canopy height. In contrast, ENV2 maintained strong correlations, with n pixels and entropy remaining strongly correlated with all yield traits (*r >* 0.77), although canopy height did not improve (*r* = 0.56 and 0.57, respectively). For both locations, the vegetation indices demonstrated poor correlations with the adjusted phenotypes. Even the TGI, which previously showed strong pairwise correlations, exhibited diminished values when experimental and environmental effects were removed (*r<* 0.45) (**Supplementary Table S6**). These findings suggest that the previously observed correlations were driven primarily by environmental influences and genotype-environment interactions (GxE), as color-based traits are inherently susceptible to variations in lighting conditions and sensor noise, compromising their reliability for genetic evaluation. These results demonstrate that ENV2 provided superior conditions for predicting conventional traits, but only when robust, non-color-based digital traits, such as pixel count and texture descriptors, were employed. The persistence of structural trait correlations using predicted BVs indicates their greater utility for genetic studies, where the goal is to capture inherent plant characteristics rather than environmentally modulated responses. Principal component analysis (PCA) of the BVs revealed similar patterns between ENV1 (**Figure 7B**) and ENV2 (**Figure 7C**), with the first two components explaining 85.3% and 85.2%, respectively. The nearly identical variance partitioning between environments indicates stable trait correlations and suggests that the underlying genetic factors controlling phenotypic variation remain consistent across different environmental conditions. Notably, vegetation indices, especially the GBVI and WI, which exhibited the lowest heritability values among the vegetation indices, formed a distinct group separated from the other traits in the PCA space.

### 3.5 Selection coincidence

**Figure 8:**
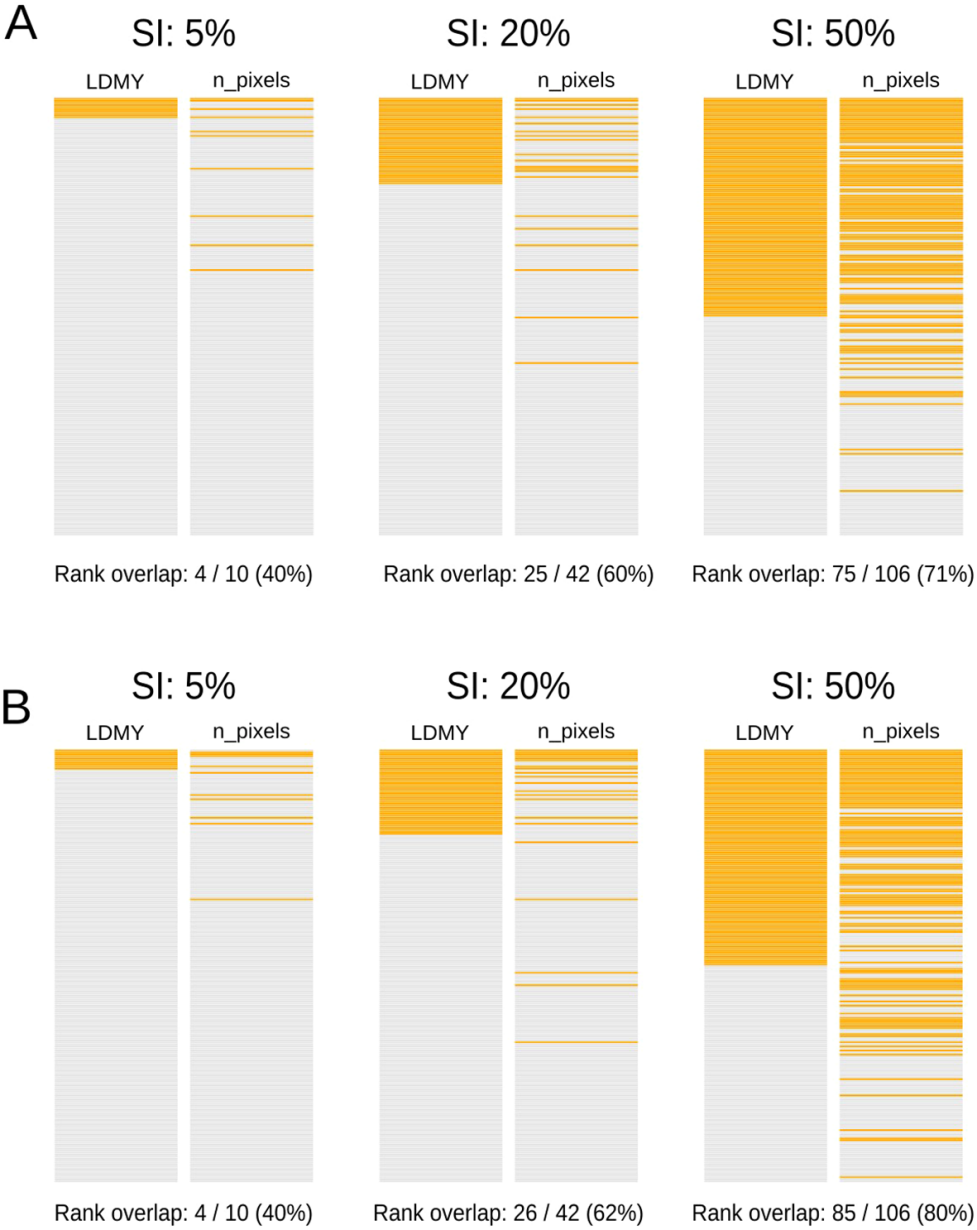
Visual comparison of the top-ranking genotypes selected by predicted breeding values (BVs). LDMY and n pixels values using mixed model effects in ENV1 **(A)** and ENV2 **(B)**. Each pair of vertical bars represents the entire population of genotypes. The orange sections (left bar) represent the genotypes ranked in 3 selection intensities (SI): 5%, 20 and 50% of genotypes for LDMY. The highlighted orange bars in the second vertical bar (right bar) of each pair show the rank position of these exact same genotypes when ranked by n pixels.

For a final, practical assessment of image-derived traits in an *M. maximus* breeding program, we compared the rankings of two key traits on the basis of their predicted BVs. The first was leaf dry matter yield (LDMY), a conventional trait for biomass production that influences the whole plant’s nutritional quality. The second was the pixel count (n pixel), an easily acquired digital phenotype that consistently demonstrated high heritability and corresponded with conventional traits. Strong and statistically significant Pearson correlations were observed between the BV rankings for LDMY and n pixel in both environments: *r* = 0.70 (ENV1) and r = 0.80 (ENV2), with *p* values less than 2.2 10-16. This finding indicates a consistent monotonic relationship between the two traits, supporting the potential of the pixel count as a proxy for LDMY in selection frameworks. Furthermore, we used a coincidence index (CI) to measure the overlap of the top-performing genotypes at three practical selection intensities. While a moderate overlap of 40-62% was observed at the more stringent 5% and 20% thresholds, a substantially higher agreement was found at the 50% selection intensity-a common threshold for early-stage breeding. Here, the overlap reached 70% in ENV1 (**Figure 8A**) and 80% in ENV2 (**Figure 8B**).

These findings provide a final validation, confirming that digital phenotyping is a reliable and efficient tool for identifying superior genotypes of *M. maximus* in early-stage selection. This is supported by the fact that a single n pixel digital assessment, performed at DAP-327 (October), produced genotype rankings with a high coincidence with those for LDMY. These LDMY ranks were based on adjusted values derived from eight conventional phenotyping dates, concluding with the final assessment at DAP-438 (February) four months later. Thus, digital phenotyping offers a significant time advantage, enabling *M. maximus* breeders to make selection decisions much earlier in the breeding cycle.

## 4 Discussion

High-throughput phenotyping (HTP) fundamentally alters plant breeding by enabling the rapid and nondestructive collection of extensive amounts of phenotypic data (Song et al., 2021; Yang et al., 2020). While the paradigm shift in data collection presents unprecedented opportunities for accelerated genetic gain, it also creates a critical gap between data acquisition and effective data-driven decision-making, emphasizing the need for new standards for data acquisition and advanced analytical strategies. This study presents a comprehensive evaluation of high-throughput digital phenotyping for *M. maximus*, a critical tropical forage grass species that feeds millions of livestock across sub-Saharan Africa, Latin America, specially Brazil, and other tropical regions. Four critical phenotypic traits for *M. maximus* breeding programs were investigated using both conventional methods and UAV image-based phenotyping. These traits include (1) green matter yield (GMY), which corresponds to total fresh plant biomass production; (2) total dry matter yield (TDMY), which measures dried water-free biomass and is directly linked to the production of nutrients important for livestock, such as proteins and fibers; (3) leaf dry matter yield (LDMY), which is obtained by separating and weighing leaves from the dried matter to estimate nutritional biomass without other plant parts, such as inflorescences, which are typically not consumed by animals and stems; and (4) canopy height (CH), which is directly linked to both biomass production and forage availability. These traits are the focus of breeding programs because they directly impact food availability for livestock production. While previous works with this species have shown promising results in predicting biomass and tillering using deep learning approaches (Santos et al., 2022; De Oliveira et al., 2021), the potential of using simpler and straightforward image-based methods, such as pixel counting, texture descriptors and vegetative indices, has yet to be explored. By systematically comparing different environments, multiple dates throughout the breeding cycle and four distinct image resolutions, we establish best practices for data collection and provide methodological guidelines that extend beyond species-specific applications to inform the implementation of digital phenotyping in forage grass and broader plant breeding programs.

### 4.1 Environmental and image resolution trade-offs in digital phenotyping

The performance disparities observed between our two experimental environments revealed critical methodological considerations for implementing digital phenotyping across repeated experimental fields. The influence of environmental conditions on remote sensing measurements is widely acknowledged (Beneduzzi et al., 2017-Jul-Aug; Haque et al., 2024; Maji et al., 2015; Ollinger, 2011; Padilla et al., 2019); however, systematic evaluations of how these factors affect digital phenotyping performance in forage crops remain limited. Many phenomics studies on crops, such as sorghum, wheat, and maize, evaluate digital traits within single environments or at single-image resolutions, with a heavy emphasis on vegetation indices, such as the NDVI and VARI. While both experimental fields we investigated enabled accurate biomass prediction, the specific digital traits providing reliable information differed substantially: vegetation indices (VIs), for example, demonstrated predictive value almost exclusively for one of the environments and only for one specific date. It should be noted that not all acquisition dates were shared across environments, which limits the scope of direct environmental comparisons and should be considered when interpreting environment-specific differences. In contrast, structural-based features, including pixel count and Haralick’s entropy, maintained consistent predictive performance across both environments and multiple measurement dates, suggesting fundamental differences in the stability and temporal robustness of information captured by RGB-based spectral versus morphological approaches under varying environmental conditions. A similar phenomenon was observed by Shu et al. (2022) in maize, where texture-based descriptors showed stronger correlations with yield traits than VIs did. These findings suggest that structural traits offer superior performance because they are directly related to the plant’s physical architecture. Entropy, for example, captures canopy complexity and leaf density, which are directly correlated with biomass accumulation. Pixel count provides a straightforward measure of plant size and canopy coverage. Variability in environmental factors, including lighting, atmospheric interference, and soil composition, directly alters leaf pigment content and spectral properties (Dhami and Cazzonelli, 2020; Esteban et al., 2015; Sims and Gamon, 2002). This environmental variability introduces complexity into image-based vegetation indices, affecting their sensitivity and accuracy in detecting vegetation health and stress because these indices respond differently to the resulting changes in color-based traits (Hernández et al., 2014; Neuwirthová et al., 2017; Wong et al., 2019). In contrast, structural characteristics are relatively more stable, making them more suitable for consistent phenotyping of yield across diverse conditions.

Another critical factor directly impacting remote sensing-derived traits is the ground sample distance (GSD), which is determined by the image acquisition distance from the ground. GSD determines image resolution and consequently affects the amount of detail acquired, as well as the computational resources required for image processing and downstream analysis. Our findings demonstrate that image resolution did not uniformly impact the correlations between digital and conventional traits. Intermediate GSDs (0.5 cm/pixel and 1.0 cm/pixel) showed stronger predictive correlations more frequently than extreme resolutions did (0.27 and 1.5 cm/pixel, respectively). The highest resolution tested (0.27 cm/pixel) exhibited the weakest correlations, likely due to increased noise in the images, such as sunlight reflecting off individual leaves and causing them to appear bleached or white. This complicates image segmentation and distorts trait measurements, highlighting a trade-off that has been noted in the literature but remains a topic for further investigation: excessively high resolution can, in some cases, reduce rather than enhance data quality (Liu et al., 2020). The statistical significance of the effects of the GSD on both pixel count and entropy across environments, as revealed by mixed-effects model analysis, confirms that resolution optimization is a critical methodological parameter that directly influences structural information extraction from aerial imagery. Importantly, we emphasize that this optimization process must be conducted for each new phenotyping scenario, including different crop species, experimental plots, environmental conditions, and sensor configurations, as the optimal GSD varies with plant architecture, canopy characteristics, and the object of interest and imaging system specifications. In this context, we also understand that conventional measurements of the traits should be performed at least once to enable the optimization process.

### 4.2 M.L for improved prediction of forage agronomic traits

The integration of image-based phenotyping and machine learning has become a powerful strategy for predicting complex agronomic traits, such as biomass yield. Our findings align with a growing body of literature demonstrating the high predictive power of machine learning models for yield estimation. For instance, studies by Feng et al. (2020) and Pranga et al. (2021) have shown random forest (RF) models and ensemble methods can achieve high accuracy (*R*^2^ *>* 0.8) for predicting yield in forage crops, such as alfalfa and perennial ryegrass. Similarly, we found that traditional machine learning models, particularly RF models, performed exceptionally well in predicting dry and fresh matter yield traits across different environments, achieving correlations with ground truth data above 0.8. Our results demonstrate that straightforward image-based features combined with classical machine learning algorithms can achieve comparable predictive performance to deep learning architectures employing convolutional neural networks and densely connected layers. While deep learning has been successfully applied to estimate biomass yield in *M. maximus* (De Oliveira et al., 2021), the implementation of complex network architectures often requires significant computational resources, technical expertise, and extensive data preparation. Given the resource constraints common in breeding programs, our finding that traditional machine learning models can achieve high predictive performance with greater accessibility and interpretability represents a critical advantage for practical implementation.

Canopy height (CH) is a key trait for forage breeders, as it often serves as a valuable proxy for biomass yield (Lübberstedt et al., 1997; Salas Fernandez et al., 2009). Traditionally, CH estimation from aerial imagery relies on 3D modeling techniques, such as structure-from-motion (SfM), which creates a digital canopy height model (CHM) from point cloud data. While highly accurate, this method is computationally intensive. Considering the substantial volume of our data and the time and effort it would take to generate the digital terrain and surface models for each orthomosaic, we decided to explore a simpler alternative: estimating CH using 2D-derived features, such as vegetation indices (VIs) and texture descriptors. Surprisingly, our approach yielded promising results. We found moderate correlations between the measured CH and VIs (0.55< *r<* 0.61), and these predictions were further improved using a multilayer perceptron (MLP) model, which achieved a mean correlation of 0.71. Notably, while the MLP model performed poorly in predicting yield values, it was the most effective for estimating CH. These results suggest that different models may be better suited for specific traits and that estimating canopy height from 2D data is a viable alternative when 3D modeling is not feasible. Further improvements could be achieved through hyperparameter optimization, a direction for future research.

To better understand our models, we conducted a feature importance analysis to identify the key drivers of trait prediction. This analysis revealed that all three classes of image-derived features-pixel counts, vegetation indices, and texture descriptors-were crucial for the predictive performance of our RF models. Specifically, five features consistently ranked as the most important predictors of forage agronomic traits across both environments: entropy, pixel count (n pixels), TGI (standard deviation), WI (standard deviation), and GBVI (standard deviation). This highlights that using multiple feature types is superior to relying on a single class of descriptors, as different features capture complementary aspects of plant architecture and physiology relevant to biomass production. For instance, spectral indices may reflect canopy greenness and photosynthetic capacity, whereas texture features capture leaf density and spatial arrangement, and structural features quantify overall plant size and growth vigor-all of which collectively contribute to forage yield. Interestingly, some of these key features showed only moderate correlations with the conventional traits on their own, yet they were vital for the predictive power of the machine learning models. This underscores how machine learning can capture nuanced phenotypic variations and complex trait interactions that are not apparent through simple correlation analysis, enabling more comprehensive assessment of yield potential than any single metric alone. While these results are promising, it is important to note that our evaluation was conducted across two environments within a single breeding cycle. The optimal image resolution (GSD) and most predictive features varied between locations, and the use of RGB sensors, while cost-effective and accessible, inherently limits spectral information compared to multispectral or hyperspectral imaging systems. Additionally, although our framework is broadly applicable, the relative performance of different feature types may vary with other forage species that have distinct canopy architectures. Despite these considerations, our findings provide a robust foundation for implementing digital phenotyping in *M. maximus* breeding, though broader validation across multiple cycles and diverse germplasm remains essential for confirming long-term selection accuracy and quantifying genetic gain.

### 4.3 Optimized phenotyping for enhanced breeding

To translate the promise of high-throughput phenotyping into actionable breeding tools, addressing the challenges of environmental heterogeneity and temporal efficiency in multiharvest forage systems is crucial. The substantial environmental variance we observed in both locations-with the interaction between block and harvest effects being significant for different traits, such as CH and GMY-underscores a well-documented challenge in tropical forage breeding: spatial and temporal field heterogeneity can obscure genetic signals and compromise selection accuracy when phenotypic evaluations rely on unadjusted plot means (Casler, 2008).

Our variance component analysis demonstrates why mixed-model approaches are not merely statistical refinements but fundamental requirements for partitioning genetic merit from microenvironmental confounding in heterogeneous field trials. Critically, the pattern of genotype-by-harvest interactions revealed important trait-specific differences in temporal stability. While biomass traits (LDMY, TDMY, and GMY) exhibited negligible genotype-by-harvest variance in ENV1 (*variance* ≈ 0) and minimal interaction in ENV2, canopy height showed substantial temporal instability in ENV2 (*variance* = 70.21, *p<* 0.001), suggesting that the genetic control of plant architecture may be more sensitive to regrowth dynamics or cutting management than to biomass accumulation. Despite these environment-specific genotype-by-harvest interaction patterns, the consistently high cross-environment heritabilities ( $H_{C}^{2}$ = 0.91-0.99 across traits) indicate that genotype rankings remain largely stable across locations, supporting the feasibility of multienvironment selection strategies in *M. maximus*.

Beyond enhancing environmental robustness, our findings underscore the significant potential for accelerated selection. We successfully leveraged digital traits derived from early postharvest imagery to predict conventional yield traits measured one month later. Most importantly, by applying a mixed-model approach to these data, we accurately ranked the top-performing genotypes with high concordance to field assessments, achieving this ranking a remarkable four months earlier than conventional methods did. This temporal advantage represents a crucial improvement in breeding efficiency, allowing for earlier selection decisions while maintaining genetic accuracy. Notably, the morphological image features n pixels and entropy exhibited not only high within-environment heritabilities but also exceptional cross-environment consistency, with values that exceeded those of some conventional measurements and most spectral indices. The negligible genotype-by-harvest variance for these structural features in both environments further supports their utility for early selection, as genetic rankings remain stable across assessment dates within locations. These findings suggest that breeding programs should implement image-based structural features in addition to spectral indices when high-throughput yield estimation protocols for *M. maximus* and potentially other tropical forage grasses are developed.

Our study also reveals important limitations of RGB-based vegetation indices (VIs) for estimating forage biomass across diverse environments and harvest cycles. While some correlations were observed at single locations and assessment dates, these relationships were inconsistent when environmental effects were statistically accounted for through mixed-model analysis. This instability likely stems from several factors that confound RGB-based spectral measurements in tropical forage systems. First, the canopy architecture in *M. maximus* changes dramatically throughout regrowth cycles-early postharvest canopies are sparse with high soil background visibility, whereas mature canopies form dense, multilayered structures that saturate RGB-based greenness indices. Second, ambient lighting conditions, cloud cover, and sun angle during image acquisition can substantially alter RGB reflectance values even when actual biomass remains constant, introducing measurement noise that obscures genetic signals; multispectral sensors incorporating NIR and red-edge bands, which are inherently less sensitive to such variation, could potentially mitigate this limitation in future studies. Third, leaf senescence, flowering, and moisture stress create temporal variations in canopy color that may not correspond proportionally to harvestable biomass, particularly when compared across different phenological stages or environmental conditions.

In conclusion, these results highlight the necessity of an integrated digital phenotyping pipeline that combines robust statistical methods with high-throughput data acquisition. For successful implementation, breeding programs should begin with a preliminary optimization phase to identify optimal image resolution and acquisition protocols for their specific conditions. This requires establishing standardized procedures that account for environmental factors and growth stage windows, alongside moderate investment in UAV equipment and training personnel in image analysis and mixed-model statistical frameworks. Our findings demonstrate that accessible machine learning algorithms can achieve high predictive accuracy without deep learning expertise, making this approach feasible for resource-constrained programs. The models presented here are supervised and therefore require conventionally measured phenotypic data as training labels, meaning some field measurement remains necessary during initial model development. Once validated, these models can reduce phenotyping effort in subsequent cycles by predicting traits directly from UAV imagery. Compared to deep learning approaches, our pipeline operates effectively at the sample sizes typical of forage breeding programs while remaining computationally accessible and producing interpretable, agronomically meaningful features. However, before fully adopting digital phenotyping for selection decisions, programs must validate the methodology within their own germplasm across multiple breeding cycles to confirm that accelerated selection maintains or improves breeding progress. The integration of such rigorous statistical and temporal strategies is therefore crucial for translating digital phenotyping into an actionable tool that accelerates plant breeding while maintaining genetic precision across diverse environmental conditions.

## Data Availability

The datasets presented in this study can be found in online repositories. The names of the repository/repositories and accession number(s) can be found below: https://data.mendeley.com/datasets/jrrb76x82h/1.

## References

[B1] Araus J. L. Kefauver S. C. Zaman-Allah M. Olsen M. S. Cairns J. E. (2018). Translating high-throughput phenotyping into genetic gain. Trends Plant Sci. 23, 451–466. doi: 10.1016/j.tplants.2018.02.001. PMID: 29555431 PMC5931794

[B2] Beneduzzi H. M. Souza E. G. Bazzi C. L. Schenatto K. (2017). Temporal variability in active reflectance sensor-measured ndvi in soybean and wheat crops. Engenharia Agrícola 37, 771–781. doi: 10.1590/1809-4430-eng.agric.v37n4p771-781/2017. PMID: 41099703

[B3] Breiman L. (2001). Random forests. Mach. Learn. 45, 5–32. doi: 10.1023/a:1010933404324. PMID: 40797221

[B4] Casler M. D. (2008). Among-and-within-family selection in eight forage grass populations. Crop Sci. 48, 434–442. doi: 10.2135/cropsci2007.05.0267

[B5] Cheng T. Zhang D. Zhang G. Wang T. Ren W. Yuan F. . (2025). High-throughput phenotyping techniques for forage: Status, bottleneck, and challenges. Artif. Intell. Agric. 15, 98–115. doi: 10.1016/j.aiia.2025.01.003. PMID: 41869561

[B6] Cristianini N. Shawe-Taylor J. (2000). An introduction to support vector machines and other kernel-based learning methods (Cambridge: Cambridge University Press).

[B7] De Oliveira G. S. Marcato Junior J. Polidoro C. Osco L. P. Siqueira H. Rodrigues L. . (2021). Convolutional neural networks to estimate dry matter yield in a Guineagrass breeding program using UAV remote sensing. 21, 3971. doi: 10.3390/s21123971, PMID: 34207543 PMC8227058

[B8] de Resende M. D. V. (2000). Análise estatística de modelos mistos via REML/BLUP na experimentação em melhoramento de plantas perenes. (Colombo, Brazil: Embrapa Florestas) p. 101

[B9] Dhami N. Cazzonelli C. I. (2020). Environmental impacts on carotenoid metabolism in leaves. Plant Growth Regul. 92, 455–477. doi: 10.1007/s10725-020-00661-w. PMID: 41868966

[B10] Dobosz B. Gozdowski D. Koronczok J. Žukovskis J. Wójcik-Gront E. (2023). Evaluation of maize crop damage using UAV-based RGB and multispectral imagery. Agriculture 13, 1627. doi: 10.3390/agriculture13081627. PMID: 41725453

[B11] Esteban R. Barrutia O. Artetxe U. Fernández-Marín B. Hernández A. García-Plazaola J. I. (2015). Internal and external factors affecting photosynthetic pigment composition in plants: A meta-analytical approach. New Phytol. 206, 268–280. doi: 10.1111/nph.13186. PMID: 25414007

[B12] Feng L. Zhang Z. Ma Y. Du Q. Williams P. Drewry J. . (2020). Alfalfa yield prediction using UAV-based hyperspectral imagery and ensemble learning. Remote Sens. 12, 2028. doi: 10.3390/rs12122028. PMID: 41725453

[B13] Freund Y. Schapire R. E. (1997). A decision-theoretic generalization of on-line learning and an application to boosting. J. Comput. Syst. Sci. 55, 119–139. doi: 10.1006/jcss.1997.1504. PMID: 39885891

[B14] García-Fernández M. Sanz-Ablanedo E. Rodríguez-Pérez J. R. (2021). High-resolution drone-acquired RGB imagery to estimate spatial grape quality variability. Agronomy 11, 655. doi: 10.3390/agronomy11040655, PMID: 30654563

[B15] Haque M. A. Reza M. N. Ali M. Karim M. R. Ahmed S. Lee K.-D. . (2024). Effects of environmental conditions on vegetation indices from multispectral images: A review. Korean J. Remote Sens. 40, 319–341. doi: 10.7780/kjrs.2024.40.4.1

[B16] Hassan M. A. Yang M. Fu L. Rasheed A. Zheng B. Xia X. . (2019). Accuracy assessment of plant height using an unmanned aerial vehicle for quantitative genomic analysis in bread wheat. Plant Methods 15, 37. doi: 10.1186/s13007-019-0419-7. PMID: 31011362 PMC6463666

[B17] Hernández E. I. Melendez-Pastor I. Navarro-Pedreño J. Gómez I. (2014). Spectral indices for the detection of salinity effects in melon plants. Sci. Agric. 71, 324–330.

[B18] Holman F. Riche A. Michalski A. Castle M. Wooster M. Hawkesford M. (2016). High throughput field phenotyping of wheat plant height and growth rate in field plot trials using UAV based remote sensing. Remote Sens. 8, 1031. doi: 10.3390/rs8121031. PMID: 41725453

[B19] Jank L. Barrios S. C. do Valle C. B. Simeão R. M. Alves G. F. (2014). The value of improved pastures to Brazilian beef production. Crop Pasture Sci. 65, 1132. doi: 10.1071/cp13319. PMID: 41161682

[B20] Jank L. Martuscello J. A. Euclides V. P. B. Braz T. G. S. Santos M. F. Resende R. M. S. . (2022). “ Panicum maximum,” in Plantas Forrageiras. Eds. da Fonseca D. M. Martuscello J. A. ( UFV), (Viçosa, Brazil: Universidade Federal de Viçosa) 122–164.

[B21] Jelihovschi E. G. Faria J. C. Allaman I. B. (2014). Scottknott: A package for performing the scott-knott clustering algorithm in R. Trends Appl. Comput. Mathematics 15, 3–17. doi: 10.5540/tema.2014.015.01.0003

[B22] Lassoued R. Macall D. M. Smyth S. J. Phillips P. W. B. Hesseln H. (2021). Data challenges for future plant gene editing: Expert opinion. Transgenic Res. 30, 765–780. doi: 10.1007/s11248-021-00264-9. PMID: 34106390 PMC8580900

[B23] Liu Y. Cen C. Che Y. Ke R. Ma Y. Ma Y. (2020). Detection of maize tassels from UAV RGB imagery with faster R-CNN. Remote Sens. 12, 338. doi: 10.3390/rs12020338. PMID: 41725453

[B24] Lübberstedt T. Melchinger A. E. Schön C. C. Utz H. F. Klein D. (1997). QTL mapping in testcrosses of European flint lines of maize: I. Comparison of different testers for forage yield traits. Crop Sci. 37, 921–931. doi: 10.2135/cropsci1997.0011183X003700030037x

[B25] Maji S. Chakraborty P. Bhowmick M. Dutta S. K. Nath R. Chakraborty P. K. (2015). Diurnal variation in spectral properties of potato under different dates of planting and N-doses. Environ. Ecol. 37, 478–473

[B26] Matese A. Di Gennaro S. (2018). Practical applications of a multisensor UAV platform based on multispectral, thermal and RGB high resolution images in precision viticulture. Agriculture 8, 116. doi: 10.3390/agriculture8070116. PMID: 41725453

[B27] Neuwirthová E. Lhotáková Z. Albrechtová J. (2017). The effect of leaf stacking on leaf reflectance and vegetation indices measured by contact probe during the season. Sensors (Basel Switzerland) 17, 1202. doi: 10.3390/s17061202, PMID: 28538685 PMC5492110

[B28] Ninomiya S. (2022). High-throughput field crop phenotyping: Current status and challenges. Breed. Sci. 72, 3–18. doi: 10.1270/jsbbs.21069. PMID: 36045897 PMC8987842

[B29] Ollinger S. V. (2011). Sources of variability in canopy reflectance and the convergent properties of plants. New Phytol. 189, 375–394. doi: 10.1111/j.1469-8137.2010.03536.x. PMID: 21083563

[B30] Padilla F. M. de Souza R. Peña-Fleitas M. T. Grasso R. Gallardo M. Thompson R. B. (2019). Influence of time of day on measurement with chlorophyll meters and canopy reflectance sensors of different crop N status. Precis. Agric. 20, 1087–1106. doi: 10.1007/s11119-019-09641-1. PMID: 41868966

[B31] Pedregosa F. Varoquaux G. Gramfort A. Michel V. Thirion B. Grisel O. . (2011). Scikit-learn: machine learning in python. J. Mach. Learn. Res. 12, 2825–2830. doi: 10.1590/1984-70332021v21Sa19, PMID: 41099703

[B32] Persa R. Ribeiro P. C. O. Jarquin D. (2021). The use of high-throughput phenotyping in genomic selection context. Crop Breed. Appl. Biotechnol. 21, e385921S6. doi: 10.1590/1984-70332021v21sa19. PMID: 41821979

[B33] Popescu M.-C. Balas V. E. Perescu-Popescu L. Mastorakis N. (2009). Multilayer perceptron and neural networks. WSEAS Trans. Cir. Sys. 8, 579–588.

[B34] Pranga J. Borra-Serrano I. Aper J. De Swaef T. Ghesquiere A. Quataert P. . (2021). Improving accuracy of herbage yield predictions in perennial ryegrass with UAV-based structural and spectral data fusion and machine learning. Remote Sens. 13, 3459. doi: 10.3390/rs13173459. PMID: 41725453

[B35] QGIS Development Team (2025). QGIS Geographic Information System.

[B36] Salas Fernandez M. G. Becraft P. W. Yin Y. Lübberstedt T. (2009). From dwarves to giants? Plant height manipulation for biomass yield. Trends Plant Sci. 14, 454–461. doi: 10.1016/j.tplants.2009.06.005. PMID: 19616467

[B37] Santos L. Junior J. M. Zamboni P. Santos M. Jank L. Campos E. . (2022). Deep learning regression approaches applied to estimate tillering in tropical forages using mobile phone images. Sensors 22, 4116. doi: 10.3390/s22114116. PMID: 35684736 PMC9185313

[B38] Schirrmann M. Giebel A. Gleiniger F. Pflanz M. Lentschke J. Dammer K.-H. (2016). Monitoring agronomic parameters of winter wheat crops with low-cost UAV imagery. Remote Sens. 8, 706. doi: 10.3390/rs8090706. PMID: 41725453

[B39] Sepúlveda-Reyes D. Ingram B. Bardeen M. Zúñiga M. Ortega-Farías S. Poblete-Echeverría C. (2016). Selecting canopy zones and thresholding approaches to assess grapevine water status by using aerial and ground-based thermal imaging. Remote Sens. 8, 822. doi: 10.3390/rs8100822. PMID: 41725453

[B40] Shakoor N. Lee S. Mockler T. C. (2017). High throughput phenotyping to accelerate crop breeding and monitoring of diseases in the field. Curr. Opin. Plant Biol. 38, 184–192. doi: 10.1016/j.pbi.2017.05.006. PMID: 28738313

[B41] Sheikh M. Iqra F. Ambreen H. Pravin K. A. Ikra M. Chung Y. S. (2024). Integrating artificial intelligence and high-throughput phenotyping for crop improvement. J. Integr. Agric. 23, 1787–1802. doi: 10.1016/j.jia.2023.10.019. PMID: 41869561

[B42] Shu M. Fei S. Zhang B. Yang X. Guo Y. Li B. . (2022). Application of UAV multisensor data and ensemble approach for high-throughput estimation of maize phenotyping traits. Plant Phenomics. 2022, 9802585. doi: 10.34133/2022/9802585. PMID: 36158531 PMC9489231

[B43] Sims D. A. Gamon J. A. (2002). Relationships between leaf pigment content and spectral reflectance across a wide range of species, leaf structures and developmental stages. Remote Sens. Environ. 81, 337–354. doi: 10.1016/s0034-4257(02)00010-x. PMID: 41810138

[B44] Sinde-González I. Gil-Docampo M. Arza-García M. Grefa-Sánchez J. Yánez-Simba D. Pérez-Guerrero P. . (2021). Biomass estimation of pasture plots with multitemporal UAV-based photogrammetric surveys. Int. J. Appl. Earth Obs. Geoinf. 101, 102355. doi: 10.1016/j.jag.2021.102355. PMID: 41869561

[B45] Song P. Wang J. Guo X. Yang W. Zhao C. (2021). High-throughput phenotyping: Breaking through the bottleneck in future crop breeding. Crop J. 9, 633–645. doi: 10.1016/j.cj.2021.03.015. PMID: 41869561

[B46] Team, R. C (2025). ScottKnott: A package for performing the Scott-Knott clustering algorithm in R (Vienna, Austria: R Foundation for Statistical Computing).

[B47] The VSNi Team (2023). Asreml: Fits linear mixed models using REML.

[B48] Wallace J. G. Rodgers-Melnick E. Buckler E. S. (2018). On the road to breeding 4.0: Unraveling the good, the bad, and the boring of crop quantitative genomics. Annu. Rev. Genet. 52, 421–444. doi: 10.1146/annurev-genet-120116-024846. PMID: 30285496

[B49] Wong C. Y. S. D’Odorico P. Bhathena Y. Arain M. A. Ensminger I. (2019). Carotenoid based vegetation indices for accurate monitoring of the phenology of photosynthesis at the leaf-scale in deciduous and evergreen trees. Remote Sens. Environ. 233, 111407. doi: 10.1016/j.rse.2019.111407. PMID: 41869561

[B50] Yang W. Feng H. Zhang X. Zhang J. Doonan J. H. Batchelor W. D. . (2020). Crop phenomics and high-throughput phenotyping: Past decades, current challenges, and future perspectives. Mol. Plant 13, 187–214. doi: 10.1016/j.molp.2020.01.008. PMID: 31981735

