# Supplementary material for: Evaluating UAV-based phenotyping strategies for Megathyrsus maximus
Source: Front Plant Sci. 2026 Apr 22;17:1798414. doi: 10.3389/fpls.2026.1798414 (PMC13143898; doi:10.3389/fpls.2026.1798414)
Supplement: Supplementary file 1 [file DataSheet1.pdf]

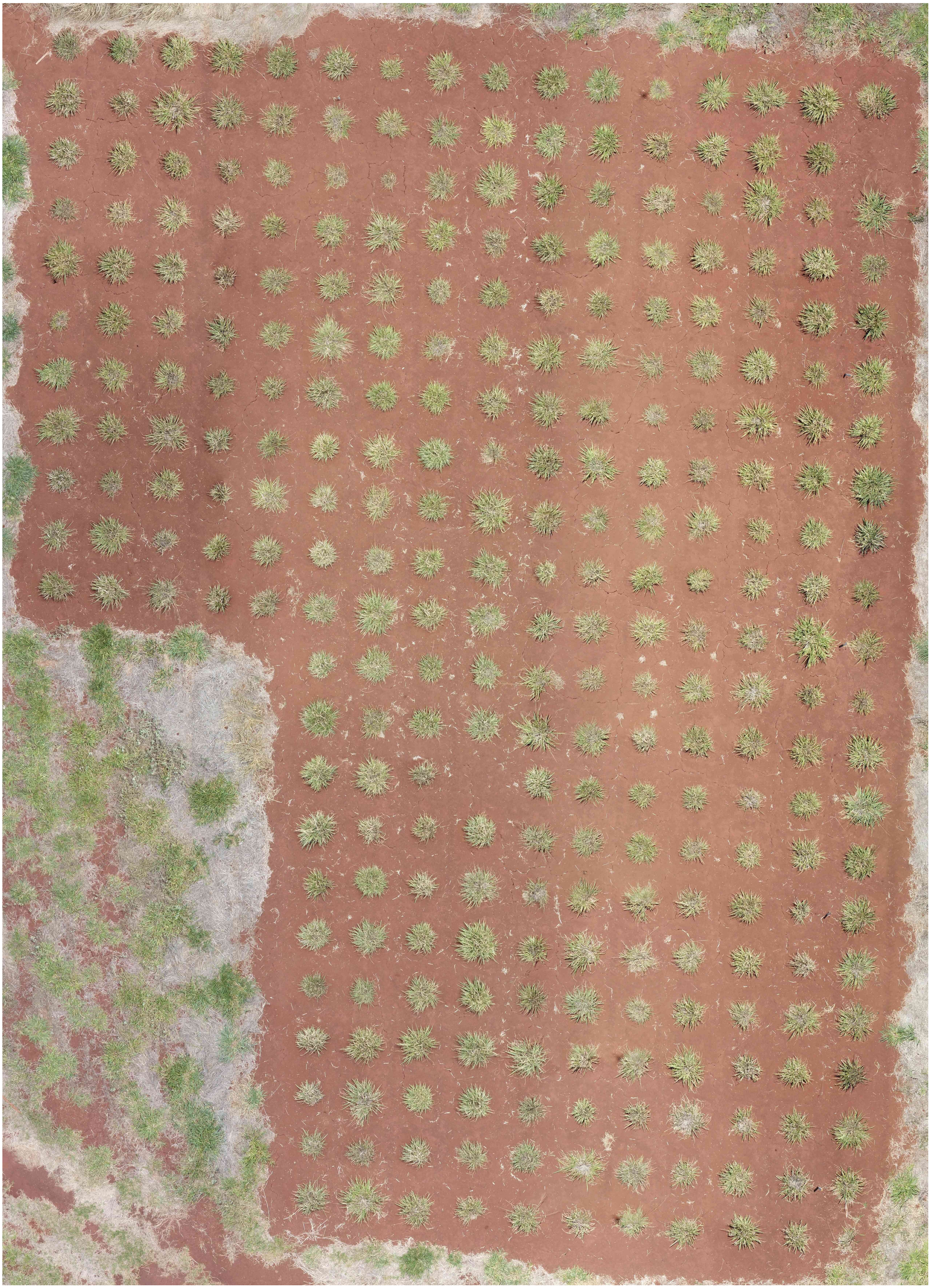

Figure S1: Example of an orthomosaic built from aerial images captured over the ENV1 plot at 327 DAP (days after planting), with a final spatial resolution of 0.5 cm/pixel. *Note: Image quality was reduced to fit publication file size constraints.*

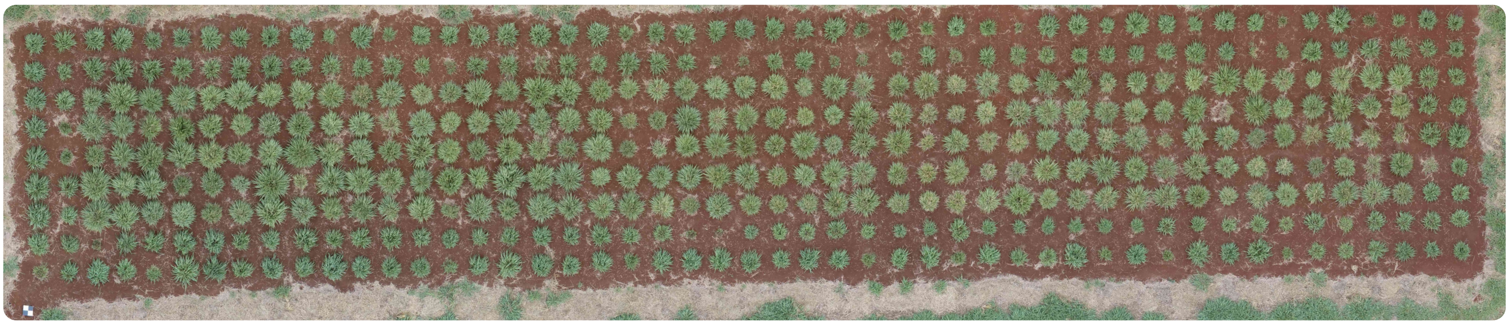

Figure S2: Example of an orthomosaic built from aerial images captured over the ENV2 plot at 327 DAP (days after planting), with a final spatial resolution of 1.0 cm/pixel. *Note: Image quality was reduced to fit publication file size constraints.*

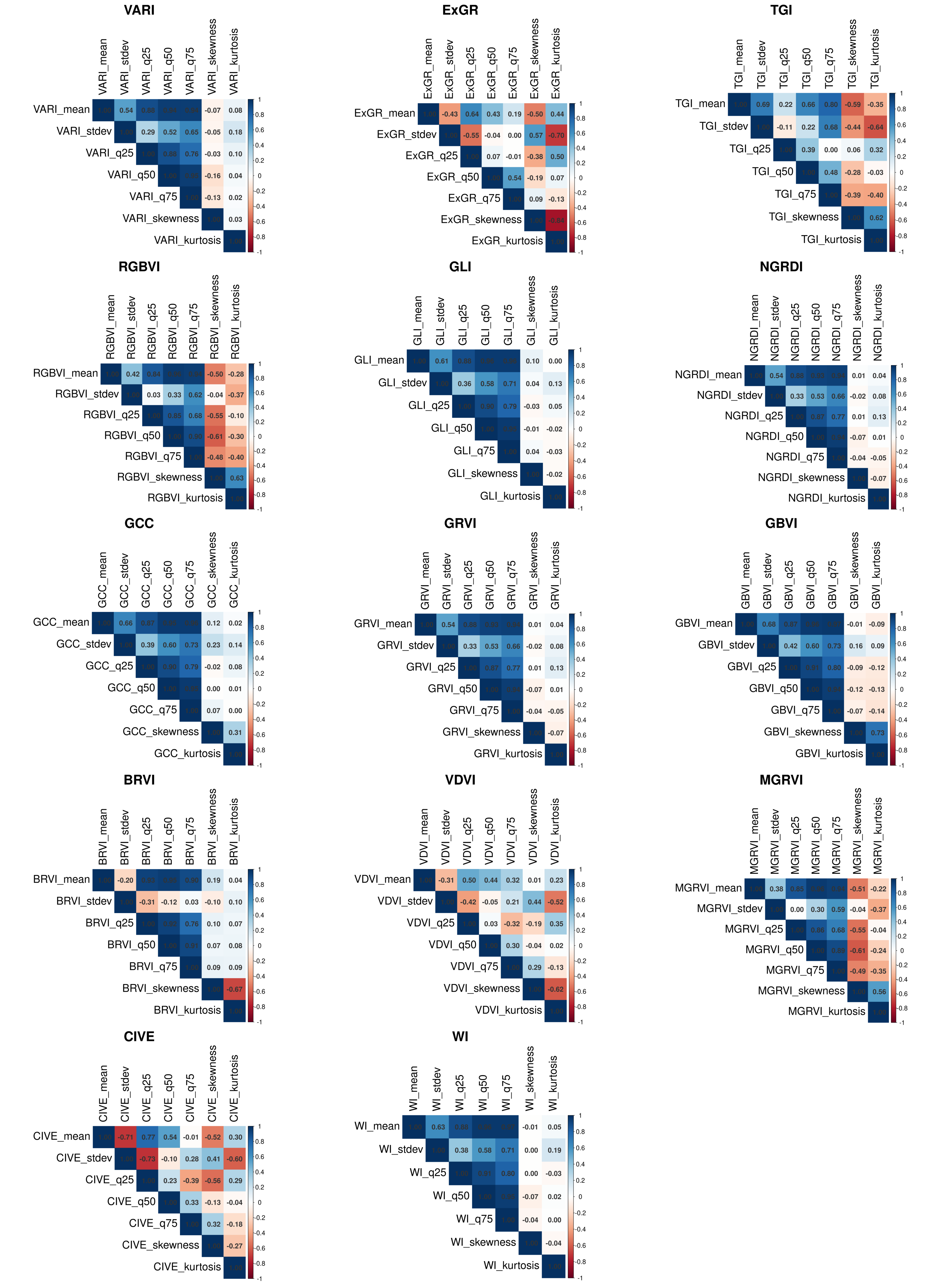

Figure S3: Correlation plots showing the Pearson's correlation coefficient (r) between different statistical metrics calculated from the Vegetation Index (VI) matrices. Each plot corresponds to a specific VI.

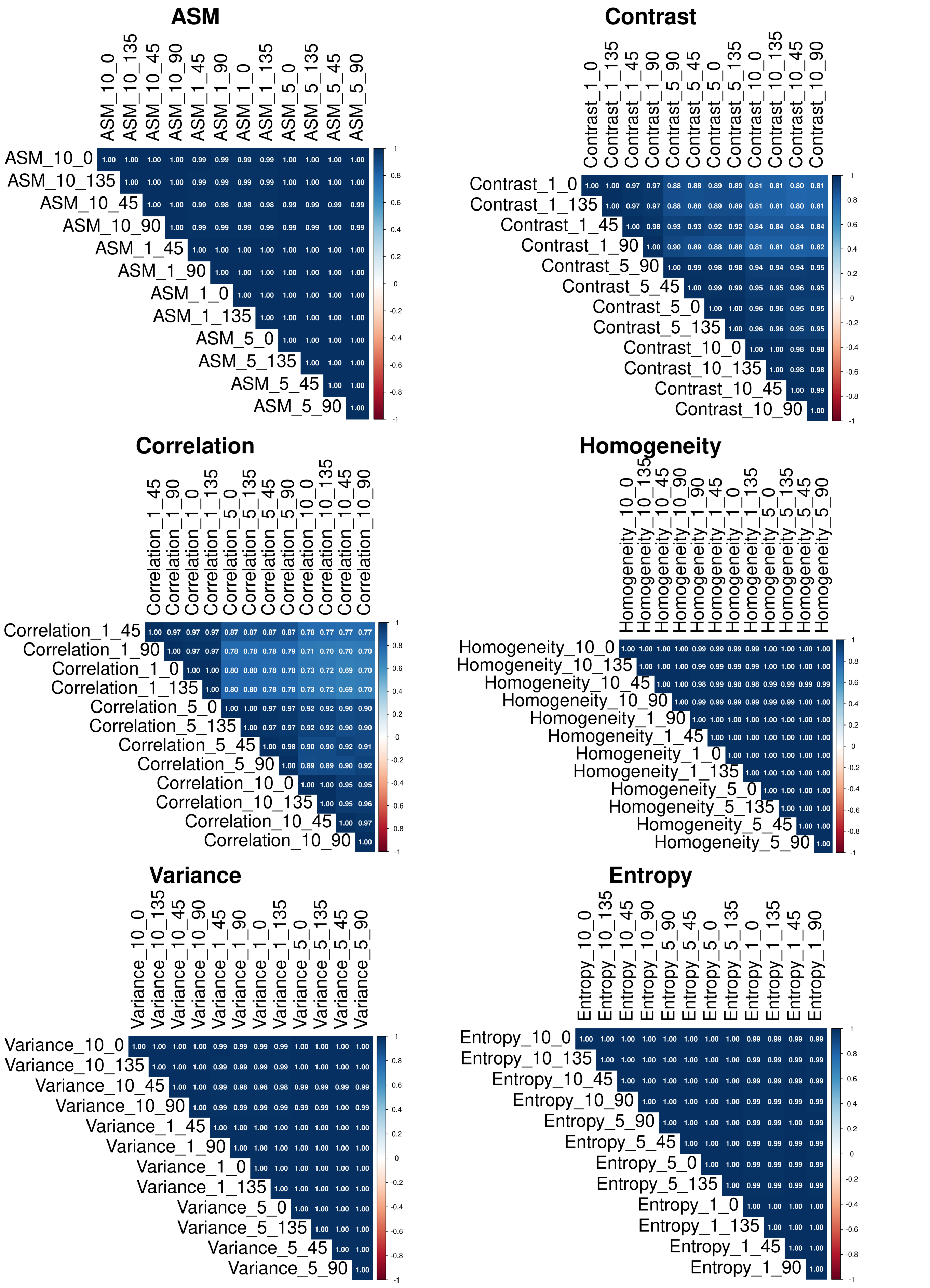

Figure S4: Correlation plots showing the Pearson's correlation coefficient (r) between values calculated using different combinations of angles and distances for each Haralick texture descriptor. Each plot corresponds to a specific Haralick descriptor.

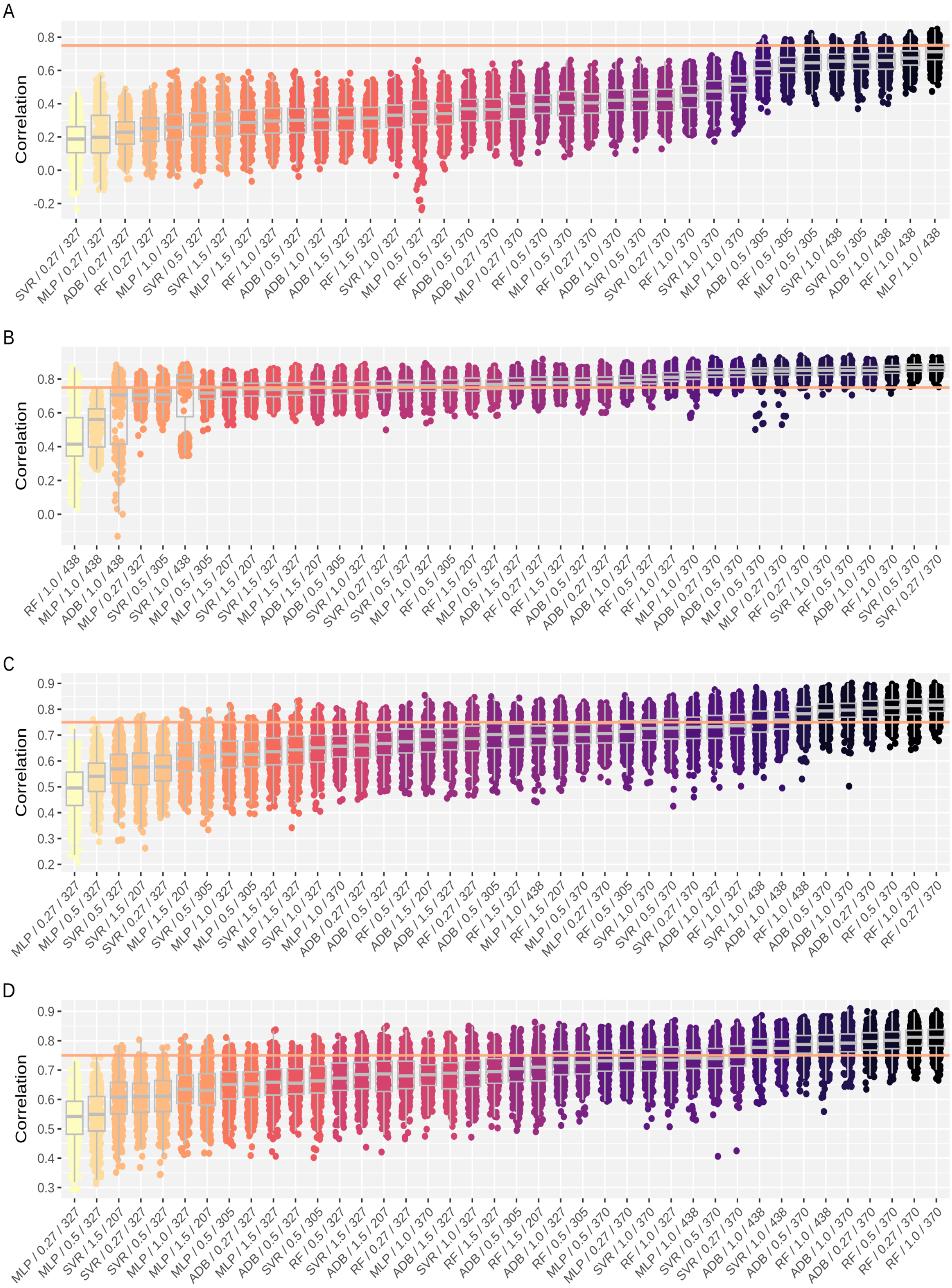

Figure S5: Performance comparison of machine learning algorithms for predicting conventional traits from remote sensing data in ENV1. Digital traits were acquired with varying combinations of dates (DAP) and ground sample distance (GSD). Only DAP showing a significant mean Pearson correlation with ground truth data for at least one conventional trait are included. A) Canopy height (CH), B) green matter yield (GMY), C) leaf dry matter yield (LDMY), and D) total dry matter yield (TDMY). Algorithms evaluated include multilayer perceptron (MLP), random forest (RF), support vector regression (SVR), and adaptive boosting (AdaBoost).

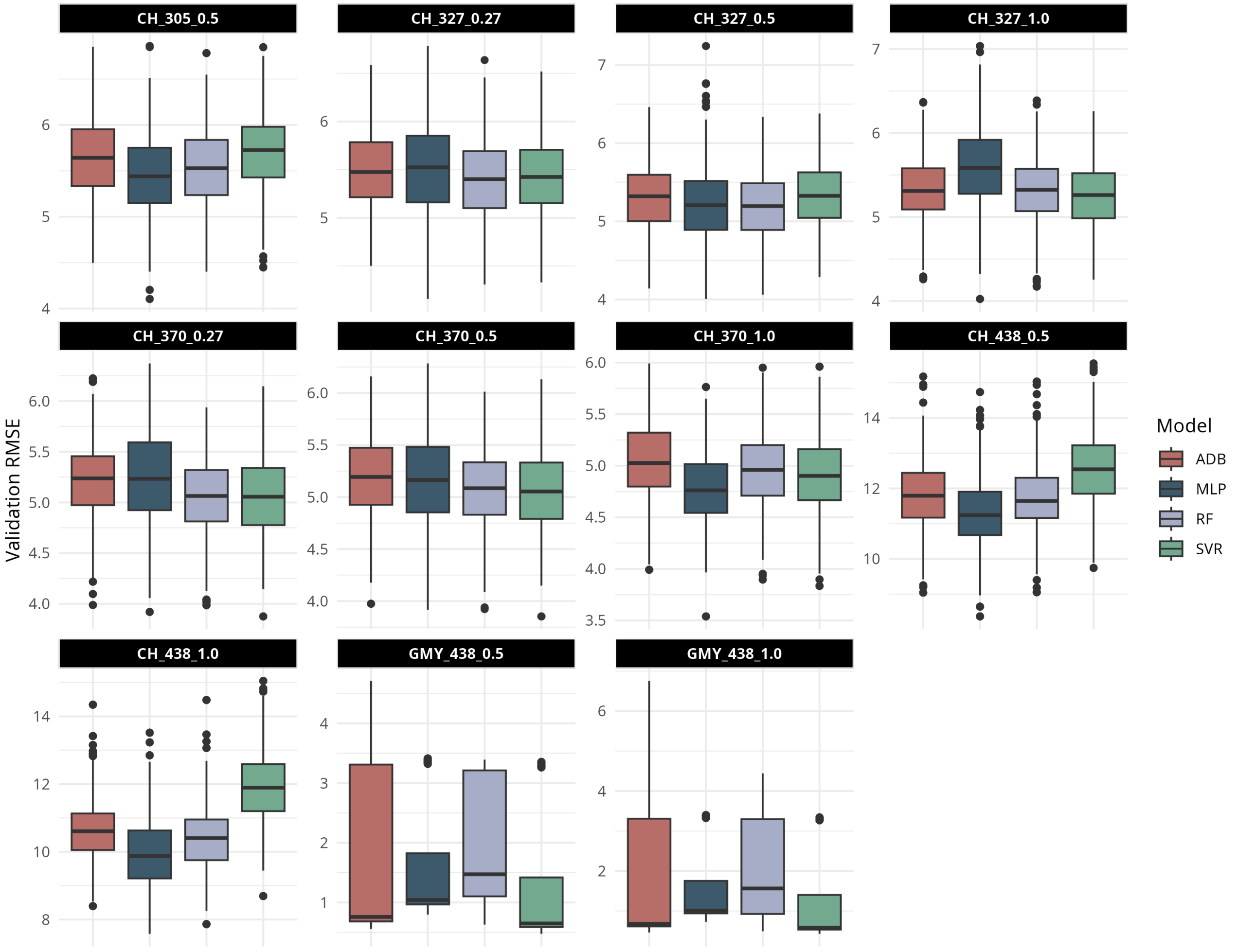

Figure S6: Box plots illustrating the distribution of Root Mean Squared Error (RMSE) for individual flights (combination of DAP + GSD) where model prediction performance was significantly different in ENV1. Only flights exhibiting a mean RMSE difference greater than 0.1 (compared across all models) and having a base prediction accuracy of r > 0.75 on the validation dataset were included. Each box plot displays the RMSE distribution across cross-fold validation runs for a specific model predicting a specific trait**.**

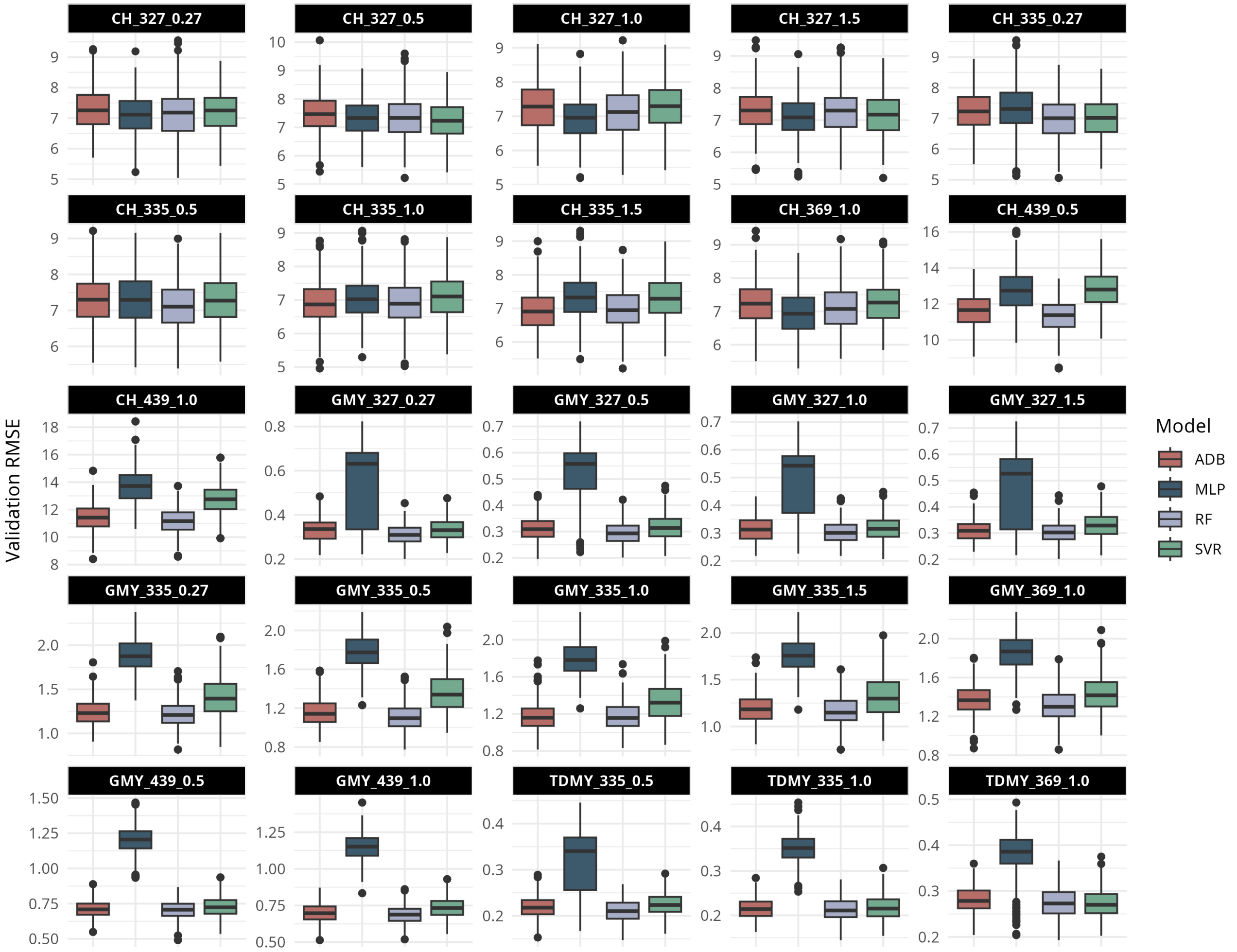

Figure S7: Box plots illustrating the distribution of Root Mean Squared Error (RMSE) for individual flights (combination of DAP + GSD) where model prediction performance was significantly different in ENV2. Only flights exhibiting a mean RMSE difference greater than 0.1 (compared across all models) and having a base prediction accuracy of r > 0.75 on the validation dataset were included. Each box plot displays the RMSE distribution across cross-fold validation runs for a specific model predicting a specific
